# Integrative Multidimensional Profiling of Individuals Recovered from Mild COVID-19 Reveals Immune–Metabolic–Oxidative Network Interactions

**DOI:** 10.3390/ijms27146518

**Published:** 2026-07-22

**Authors:** Iole Macchia, Valentina La Sorsa, Francesca Marcon, Cristina Andreoli, Alessandro Giuliani, Donatella Pietraforte, Maria Cristina Quattrini, Egidio Iorio, Mattea Chirico, Maria Elena Pisanu, Enrica Montefiore, Francesca Luciani, Antonio Martina, Fabiola Mancini, Martina Borghi, Valentina Durastanti, Maria Concetta Altavista, Francesca Urbani

**Affiliations:** 1 Oncology and Molecular Medicine Department, National Institute of Health, 00161 Rome, Italy; iole.macchia@iss.it; 2 Research Promotion and Coordination Service, National Institute of Health, 00161 Rome, Italy; valentina.lasorsa@iss.it; 3 Environment and Health Department, National Institute of Health, 00161 Rome, Italy; francesca.marcon@iss.it (F.M.); cristina.andreoli@iss.it (C.A.); 4 Independent Researcher, 00183 Rome, Italy; alessandrogiuliani480@gmail.com; 5 Core Facilities, National Institute of Health, 00161 Rome, Italy; donatella.pietraforte@iss.it (D.P.); mariacristina.quattrini@iss.it (M.C.Q.); egidio.iorio@iss.it (E.I.); mattea.chirico@iss.it (M.C.); mariaelena.pisanu@iss.it (M.E.P.); enrica.montefiore@iss.it (E.M.); 6 National Center for the Control and Evaluation of Medicines, National Institute of Health, 00161 Rome, Italy; francesca.luciani@iss.it (F.L.); antonio.martina@iss.it (A.M.); 7 Infectious Disease Department, National Institute of Health, 00161 Rome, Italy; fabiola.mancini@iss.it (F.M.); martina.borghi@iss.it (M.B.); 8 Neurology Unit, San Filippo Neri Hospital, Rome 1 Local Health Authority (ASL RM1), 00135 Rome, Italy; valentina.durastanti@aslroma1.it (V.D.); mariac.altavista@aslroma1.it (M.C.A.)

**Keywords:** COVID-19, vaccination, leukocytes, oxidative stress, metabolomics, lipidomics, DNA damage, network analysis, flow cytometry, immunophenotyping

## Abstract

The COVID-19 pandemic underscored the need to better characterize immune and molecular responses following SARS-CoV-2 infection and vaccination. Beyond antibody and cellular immunity, COVID-19 involves oxidative stress and DNA damage, affecting repair mechanisms and metabolic adaptation linked to immune resilience. Here, we present a multidimensional analysis of 20 individuals who recovered from mild COVID-19, integrating clinical features with humoral and cellular immune responses, T cell and myeloid phenotypes, oxidative stress, DNA damage, and metabolomic and lipidomic profiles. Although most individual parameters fell within physiological ranges, network modeling revealed structured associations spanning multiple biological domains. A central finding was a coherent cluster organized around vaccine dose number, linking anti-Spike antibody titers, oxidative stress, bioenergetic signatures, and granulocyte activation. Higher vaccination was associated with stronger humoral responses, lower oxidative stress, and a more balanced myeloid–metabolic profile, suggesting a potential protective role extending beyond antibody induction. Additional associations linked symptom patterns to T cell differentiation states, anti-nucleocapsid responses to systemic inflammation, and anaerobic signatures to DNA damage markers, revealing interconnections between immunometabolism, clinical expression, and genomic stress. Despite the small sample size, these findings offer a preliminary systems-level perspective on mild COVID-19 recovery and illustrate the value of integrative exploratory frameworks in infectious disease research, laying the groundwork for validation in larger longitudinal cohorts.

## 1. Introduction

The COVID-19 pandemic highlighted the complexity of host immune responses to SARS-CoV-2 infection and vaccination, involving a dynamic interplay between innate and adaptive immune pathways [1,2]. Even mild infection can induce long-term immunological changes, including modulation of antibody production, cellular immunity, and immune cell phenotypes, collectively shaping protection against reinfection and severe disease [3]. The peripheral immunophenotype represents a key indicator of immune competence [4], and rare T-cell populations—including double-positive (DP), double-negative (DN), and Vδ2 γδ T cells—contribute to antiviral cellular responses in ways that are influenced by age, vaccination history, and other host factors [5]. SARS-CoV-2 infection is accompanied by changes in blood innate immune cell numbers and phenotypes, with potential implications for the severity and resolution of COVID-19 [6]. Among innate immune cells, monocyte subsets (classical, intermediate, and non-classical) display distinct inflammatory activation patterns associated with disease severity [7,8], while eosinophils undergo dynamic phenotypic changes during infection, with surface markers such as CD11b, CD62L, and PD-L1 emerging as potential prognostic biomarkers [9,10].

Oxidative stress is a central feature of SARS-CoV-2 pathophysiology, contributing to immune dysregulation and disease severity [11,12]. Elevated levels of lipid oxidation products, such as thiobarbituric acid (TBA) reactive substances (TBARS), as well as protein oxidation products, such as carbonyl groups, have been documented in COVID-19 patients [13], alongside markers of DNA damage correlating with clinical severity [14]. Virus-induced oxidative stress may impair DNA repair mechanisms, promoting genomic instability as evidenced by micronuclei formation, DNA repair foci, and increased comet assay tail moments [15]. TBARS levels have been associated with disease severity, showing higher values in severe cases and decreasing following vaccination or recovery [16]. Age-related increases in oxidative stress have been associated with reduced vaccine-induced immune responses [17], while evidence of vaccine-related oxidative effects remains heterogeneous—ranging from transient reactive oxygen species increases following mRNA vaccination [18] to oxidative stress markers in rare inflammatory events such as myopericarditis [19]. Together, these observations underscore the need to characterize the molecular links between oxidative burden and immune outcomes across different exposure histories [20].

SARS-CoV-2 infection is also characterized by substantial metabolic remodeling. Metabolomic and lipidomic studies have identified disease-relevant signatures, including glutamate and kynurenine as markers of severity and patient stratification [21,22,23,24]. Notably, plasma lipid profiles influence T lymphocyte activation, differentiation, and memory formation, as well as granulocyte inflammatory responses [25], establishing a functional link between systemic metabolism and immune regulation. Beyond their structural and energetic roles, metabolites—including plasma lipids, glucose, amino acids, and microbiota-derived short-chain fatty acids—act as direct immunological regulators. They govern T lymphocyte activation, effector differentiation, and memory formation through mTOR/AMPK metabolic checkpoints, IDO-mediated kynurenine immunosuppression, and epigenetic reprogramming, while also shaping innate immune responses. The interplay among oxidative stress, DNA damage, and metabolic adaptation thus represents a key—and still incompletely understood—axis of COVID-19 pathophysiology [15].

In a previous study [26], we characterized SARS-CoV-2-derived HLA-A*02:01-restricted peptides capable of eliciting CD8+ T cell responses in vaccinated individuals who had also recovered from mild COVID-19, revealing associations between peptide-specific cellular immunity, humoral responses, and host factors such as age and vaccination history. Vδ2 γδ and double-negative T cells emerged as candidate biomarkers of immune protection. Building on these findings, the present study extends the analysis to a larger cohort including both HLA-A*02-positive and HLA-A*02-negative individuals through the integration of immunophenotypic, metabolomic, lipidomic, oxidative stress, and DNA damage profiling. The primary objective was to identify coordinated immune–metabolic–oxidative signatures associated with recovery from mild COVID-19 by integrating multiple biological dimensions within the same individuals through a systems-level, network-based analytical approach. A secondary, methodological objective was to evaluate the validity and informative value of a comprehensive multidimensional framework—combining clinical, lifestyle, immunological, metabolomic, lipidomic, oxidative stress, and genomic integrity parameters—as a hypothesis-generating strategy in exploratory studies of host responses to infectious diseases, and specifically to assess whether such an approach reveals biologically meaningful patterns of association that remain undetectable using conventional univariate analyses. Rather than identifying immediately applicable clinical biomarkers, this exploratory study was designed to generate hypotheses and establish an integrative analytical framework that could inform the prioritization of candidate signatures for validation in larger prospective cohorts, with the longer-term goal of supporting patient stratification and personalized preventive or therapeutic strategies.

## 2. Results

### 2.1. Subjects’ Characteristics

**Figure 1:**
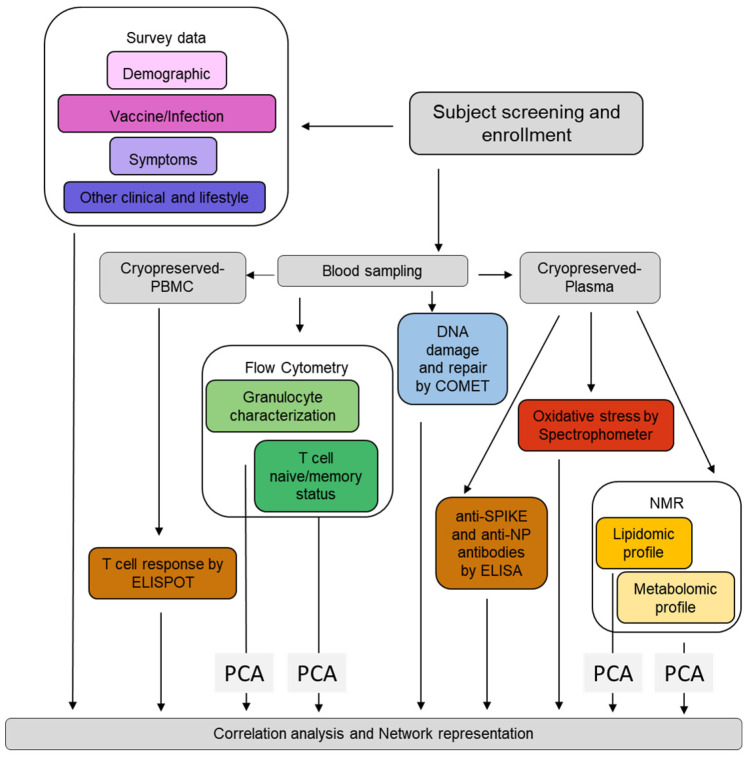
Experimental workflow.

An overview of the study design is provided in Figure 1. The study cohort expanded upon that described in our previous work [26], to which the reader is referred for full methodological details. Overall, twenty healthy subjects recovered from COVID-19 were enrolled between 19 November 2021 and 19 December 2022, with infections occurring between 23 July 2021 and 2 May 2022; the study cut-off date was 21 December 2022. Among the 20 participants, 15 were HLA-A*02-positive (14 reported in the first study plus one additional HLA-A*02-positive subject) and five were HLA-A*02-negative subjects.

Table 1 summarizes the main demographic characteristics (sex at birth—hereafter referred to as sex—and age); vaccination history (dose number); and infection-related details, including the date of the first positive swab, the probable infecting SARS-CoV-2 variant of concern (VOC), the total number of symptoms, and the following calculated time intervals (ΔT), calculated in days between positivity and sampling date (PS-ΔT), vaccination and sampling date (VS-ΔT), vaccination and positivity date (VP-ΔT), positivity and negativization date (PN-ΔT), positivity and cut-off date (PCO-ΔT), and sampling and cut-off date (SCO-ΔT).

Subjects were arranged in Table 1 according to the date of their first positive SARS-CoV-2 test.

The cohort comprised 14 female and 6 male subjects, with a median age of 50 years (range: 18–69 years). Based on infection timing and epidemiological data, 6 subjects were presumably infected with the Delta variant, 12 with Omicron BA.1, and 2 with Omicron BA.2. All participants experienced a paucisymptomatic or mild COVID-19 course, reporting a median of 5 symptoms (range: 1–13). Two subjects were unvaccinated, 4 received one vaccine dose, 4 received two doses, and 10 received three doses prior to infection. Vaccination schedules were heterogeneous, encompassing single-dose regimens (Johnson & Johnson, Moderna and Pfizer) as well as mixed combinations across two or three doses of different brands. Median values of all ΔT intervals, along with their minimum and maximum values, are reported in Table 1.

#### 2.1.1. Symptom-Based Characterization of Subjects

Supplementary Table S1 displays the symptoms reported by the 20 participants during their mild COVID-19 infection. The most frequently reported symptoms were malaise/fatigue and fever (12/20), followed by headache (10/20); cough, throat pain, rhinorrhea, joint pain (8/20); muscle pain (7/20); ageusia (6/20); and anosmia (4/20). Confusional state, shortness of breath, thoracic pain, and rapid pulse were each observed in 3/20 subjects, while diarrhea and conjunctivitis occurred in 2/20. A single patient reported each of the following: oxygen saturation below 96%, nausea/vomiting, rash, abdominal pain, paresthesia, and dental/mandibular pain. No subject presented with inability to walk, wheezing, bleeding, lymphadenopathy, seizures, lower chest indrawing, or skin ulcer.

#### 2.1.2. Other Clinical and Lifestyle Characteristics

Additional features potentially contributing to individual profiles were collected via survey (Supplementary Table S2). Among the 20 participants, 14 used concomitant therapy during COVID-19 infection, 11 reported X-ray exposure in the preceding year, 8 had pre-existing medical conditions, 8 received an influenza vaccine in the preceding year, 5 took paracetamol during infection, 5 used ibuprofen during infection, 4 were mild/light habitual alcohol consumers, 4 reported allergies, 4 were habitual tobacco smokers, 3 used *N*-acetylcysteine during infection, 3 had a history of previous infectious diseases, and 3 engaged in intensive sport activity.

### 2.2. SARS-CoV-2–Specific Antibody Titers in Plasma

Individual ELISA results for anti-Spike and anti-nucleoprotein (anti-NP) antibody levels are presented as line graphs in Supplementary Figure S1A. Consistent with our previous findings [26], all participants exhibited anti-Spike antibody levels well above the positivity threshold of 0.8 U/mL, with a median of 36,415.00 U/mL (range: 3.80–105,300.00 U/mL). The two lowest values were observed in the unvaccinated subjects: Subject 17 showed the minimum value of 3.80 U/mL and Subject 3 showed 48.60 U/mL, both nonetheless above the cut-off. Anti-NP antibody levels, though generally lower in magnitude compared to anti-Spike levels, were above the positivity threshold of 1 cut-off index (COI) in nearly all subjects, with a median of 17.48 COI (range: 0.73–285.50 COI), except for Subject 9, whose levels fell below the threshold.

### 2.3. Assessment of SARS-CoV-2–Specific T Cell Immune Responses

T cell responses to the Peptivator S and Peptivator N peptide pools were assessed by interferon gamma (IFN-γ) ELISpot in cryopreserved peripheral blood mononuclear cells (cPBMCs) from all 20 subjects (Supplementary Figure S1B); in HLA-A*02:01–positive samples (15 subjects), responses to the previously characterized immunogenic peptides Spike LA-9 and Spike KL-9 [26], as well as to the cytomegalovirus/Epstein–Barr virus/influenza virus (CEF) positive control pool, were additionally evaluated (Supplementary Figure S1C).

Subjects exhibited variable responses across all stimulations. Median Spot-Forming Cell (SFC) indices (SFCI—with ranges) were as follows: 9.45 (1.50–43.80) for Peptivator S; 2.75 (0.00–25.00) for Peptivator N; 2.00 (1.00–23.00) for LA-9; 6.10 (0.30–32.00) for KL-9; and 13.00 (1.24–287.30) for the CEF peptide pool. Only one unvaccinated subject (Subject 3) fell below the arbitrary positivity threshold of 2 SFC for Peptivator S, while some subjects with more recent infections showed notably higher responses to this stimulus (Subjects 13-17-18). For Peptivator N, 6 out of 20 subjects were below threshold, with generally lower response magnitudes compared to Peptivator S. The CEF pool elicited robust responses in all but one subject (Subject 7). For the individual peptides, 3 out of 15 subjects were negative for KL-9 and 7 out of 15 for LA-9. This variability underscores the heterogeneity of T cell memory following SARS-CoV-2 infection and/or vaccination, with some individuals responding robustly across multiple stimuli and others showing more selective or lower-magnitude responses.

A positive correlation was observed between ELISPOT responses to Peptivator S and Peptivator N (Spearman’s ρ = 0.572, *p* = 0.026), as well as between Peptivator S and the CEF pool (Spearman’s ρ = 0.644, *p* = 0.002), suggesting that the magnitude of the SARS-CoV-2–specific T cell response may reflect a generally robust cellular immune competence. Conversely, a negative correlation was observed between anti-NP antibody levels and T cell responses to the KL-9 peptide (Spearman’s ρ = −0.579, *p* = 0.024), indicating a potential inverse relationship between humoral immunity against NP and cellular immunity directed against this specific Spike epitope.

### 2.4. Peripheral Blood T Cell Memory Profile Characterization

The naïve/memory status of both major (total CD3^+^, CD4^+^, CD8^+^) and unconventional/minor (DP1, DP2, DN, and Vδ2 γδ) T cell subpopulations was assessed by immunophenotyping of fresh whole blood using a 7-color multiparametric flow cytometry (MFC) panel in a subset of 18 subjects (Supplementary Table S3a and Figure S2).

As shown in Supplementary Figure S3, naïve cells predominated within the total CD3^+^ and CD4^+^ compartments, whereas CD8+ T cells displayed a higher frequency of terminally differentiated (TD) cells. Among minor subsets, DP2, DN and Vδ2 γδ T cells predominantly exhibited TD or EM phenotypes. The constitutively low CCR7 expression in Vδ2 T cells resulted in the expected reduced representation of naïve and central memory (CM) populations within this subset. DP1 cells were mainly CM and effector memory (EM). These results confirm distinct distributions of naïve and memory subsets across both major and minor T cell populations following COVID-19, consistent with our previous findings in a subgroup of subjects [26].

**Figure 2:**
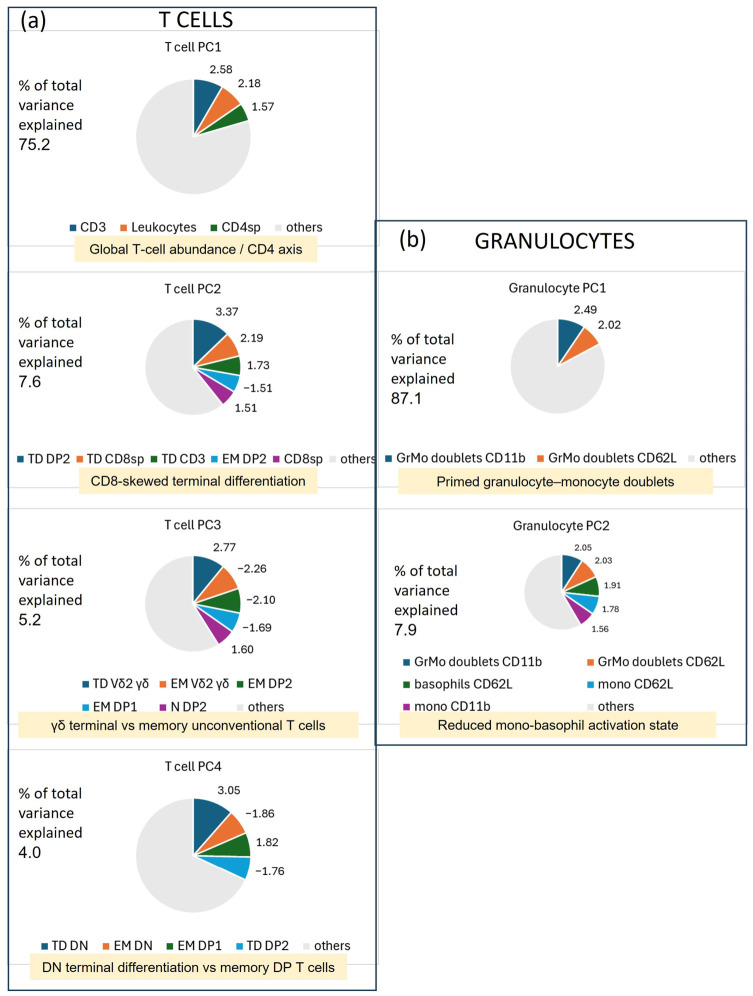
Principal component analysis of peripheral blood T cell (**a**) and granulocyte/monocyte (**b**) subsets. Pie charts show the relative contribution (score coefficients) of individual cell subsets to each principal component (PC). Each PC was assigned a biological label reflecting the dominant immunological theme captured by its leading variables.

To identify common patterns of interindividual variability and reduce dimensionality for downstream correlation analyses, PCA was applied to a transposed dataset (see Section 4.12 and Figure 2a). Four principal components (PCs) were extracted, collectively explaining 92.0% of total variance. Based on the preponderance of the leading variables within each component, we assigned a biological label to each PC to facilitate interpretation. PC1 (75.2% of the total explained variance), dominated by CD3^+^ total T cells, total leukocytes, and CD4sp T cells, was interpreted as reflecting natural interindividual variability in the major T cell compartments and labeled “Global T-cell abundance/CD4 axis”. PC2 (7.6%), primarily driven by terminally differentiated DP2, CD8sp and CD3^+^ T cells, in contrast to EM DP2 T cells, was interpreted as capturing differences in cytotoxic effector orientation across subjects and labeled “CD8-skewed terminal differentiation”. PC3 (5.2%), characterized by the opposing contributions of terminally differentiated Vδ2^+^ γδ and N DP2 T cells against EM Vδ2^+^ γδ, DP1 and EM DP2 cells, was interpreted as reflecting variation in the relative balance between γδ and double-positive subsets within the unconventional T cell compartment and labeled “γδ terminal vs. memory unconventional cells”. PC4 (4.0%), contrasting TD DN T cells and EM DP1 with EM DN and TD DP2 cells, was interpreted as capturing differences in the relative contribution of double-negative versus double-positive subsets within the unconventional T cell compartment and labeled “DN terminal differentiation vs. memory DP T cells”. Complete PC scores are reported in Supplementary Table S5.

### 2.5. Peripheral Blood Granulocyte and Monocyte Profile Characterization

Circulating innate immune cells were characterized by flow cytometric analysis of granulocyte and monocyte subsets in fresh peripheral blood (Supplementary Table S3b and Figure S4). The identified populations comprised neutrophils, eosinophils, basophils, and monocytes—the latter further subdivided into classical, non-classical, and granulocyte–monocyte (Gr–Mo) doublets. Surface expression of CD11b (activation marker), PD-L1/CD274 (immune checkpoint marker), and CD62L (adhesion molecule) was assessed as mean fluorescence intensity (MFI), and the frequency of eosinophils and basophils within the CD294+ gate was additionally evaluated, yielding 31 parameters across a subset of 13 subjects.

The overall distribution of myeloid–monocytic subpopulations (Supplementary Figure S5) was consistent with patterns typically observed in healthy adults. Neutrophils were the predominant population; eosinophils accounted for a small fraction, with basophils constituting an even smaller subset. Within the monocyte compartment, classical monocytes were the most abundant subset, followed by Gr–Mo doublets, with non-classical monocytes representing the least abundant population.

Regarding activation marker expression, CD11b showed overall higher levels within the monocytic compartment. CD274 was more pronounced in non-classical monocytes and Gr–Mo doublets, while CD62L was minimally expressed in eosinophils but markedly elevated in Gr–Mo doublets. Notably, Gr–Mo doublets exhibited the highest co-expression of CD11b, CD274, and CD62L across all subsets, suggesting a distinct activation or differentiation state within this population.

PCA was similarly applied to the transposed granulocyte/monocyte panel dataset (Figure 2b). Two principal components were extracted, collectively explaining 94.8% of total variance. Based on the preponderance of the leading variables within each component, we assigned a biological label to each PC to facilitate interpretation. PC1 (87.1% of the total explained variance), dominated by Gr–Mo doublets with high CD11b and CD62L expression, was interpreted as reflecting a primed but not fully activated state of the myeloid–monocytic compartment—consistent with the role of CD11b in adhesion and migration and the known downregulation of CD62L upon cell activation—and labeled “Primed granulocyte–monocyte doublets”. PC2 (7.7%), characterized by the contributions of Gr–Mo doublets CD62L, basophils CD62L, monocyte CD62L, and monocyte CD11b, was interpreted as reflecting a state of reduced activation across both monocytic and basophilic compartments and labeled “Reduced mono-basophil activation state”. Complete PC scores are reported in Supplementary Table S6. Together, these findings suggest that interindividual differences in baseline monocyte activation and priming states underlie the observed variability, with Gr–Mo doublets acting as sensitive reporters of subtle shifts in the broader myeloid activation landscape.

### 2.6. Metabolic and Lipid Profiles

**Figure 3:**
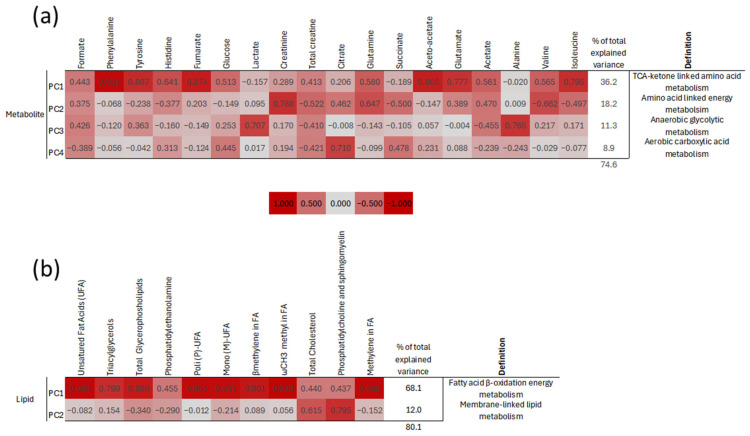
Principal component analysis of plasma metabolomic (**a**) and lipidomic (**b**) profiles. Heatmaps show the loading coefficients of individual analytes on each PC. Each PC was assigned a functional label reflecting the predominant metabolic or lipidomic pathway captured by its leading variables; pathway annotations are shown on the right.

Plasma metabolite and lipid profiles were assessed in the whole cohort of 20 subjects and were overall consistent with patterns typically observed in healthy adults (Supplementary Figure S6), though with heterogeneous distributions across subjects and partially overlapping trends for selected markers. Given the large number of biochemical parameters assessed, PCA was applied to reduce dimensionality and identify underlying patterns of variation (Figure 3), enhancing the robustness of subsequent integrative analyses.

For the metabolite dataset, four PCs explaining 74.6% of total variance were extracted. PC1 (36.2%) was enriched in metabolites indicative of “Tricarboxylic Acid (TCA)-ketone linked amino acid metabolism”. PC2 (18.2%) appeared to reflect an “amino acid linked energy metabolism”. PC3 (11.3%) was associated with “anaerobic glycolytic metabolism”, while PC4 (8.9%) showed a pattern consistent with “aerobic carbonyl acid metabolism”. For the lipid dataset, two PCs explaining 80.1% of total variance were extracted. PC1 (68.1%) was enriched in lipid species suggestive of “fatty acid β-oxidation energy metabolism”, while PC2 (12.0%) reflected lipid species involved in cellular membrane metabolism and was labeled as “membrane-linked lipid metabolism”.

### 2.7. Oxidative Stress Markers

Plasma levels of the two established oxidative stress biomarkers reflecting lipid and protein oxidation, i.e., TBARS and protein carbonyl groups, respectively, were measured in all enrolled subjects and visualized as line plots (Supplementary Figure S7A). Both markers showed interindividual variability, with levels falling within ranges typically reported in healthy adult populations. Protein carbonyl levels displayed marked variability across subjects, whereas TBARS showed a less heterogeneous distribution. Notably, elevated carbonyl group levels were observed in individuals infected at earlier time points (Subjects 1–5), while lower levels were detected in those more recently infected (Subjects 18–20).

### 2.8. DNA Damage and Repair Kinetics

Levels of DNA damage and the repair capacity were assessed in blood samples of a subgroup of 10 participants in the study by applying the comet assay. The spontaneous level of DNA strand breaks (%TI), representing baseline DNA damage, showed low interindividual variability, whereas the percentage of residual DNA damage (%RD) after 15 and 30 min of repair (denoted as %RD15 and %RD30, respectively) displayed markedly higher interindividual variability. As shown in Supplementary Figure S7B, %RD15 and %RD30 were strongly correlated and showed a decreasing tendency in individuals with more recent infection, despite substantial interindividual differences being observed. Overall, the values observed for these parameters were generally within those typically reported in healthy adult populations.

### 2.9. Integrative Network Analysis Reveals Coordinated Biological and Clinical Patterns

**Figure 4:**
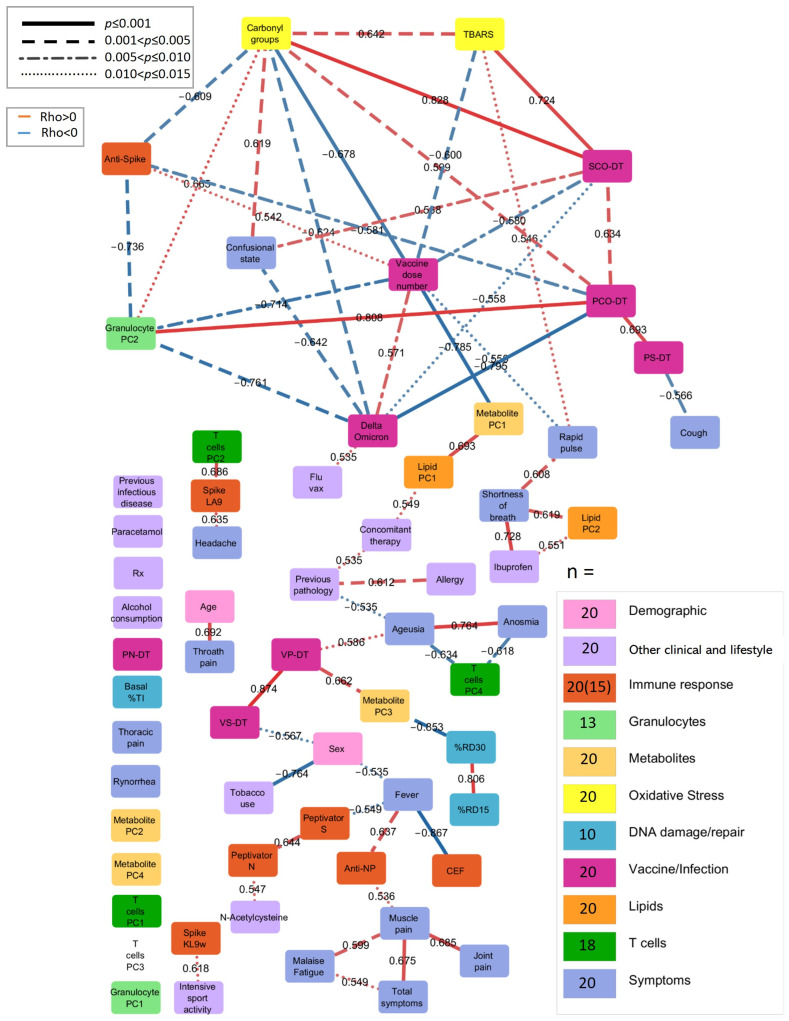
Correlation network among study parameters. Statistically significant Spearman correlations (*p* ≤ 0.015) among all study variables are shown. Nodes are color-coded by variable category (see legend). Edges represent Spearman’s Rho correlation coefficients. Rho values are reported numerically at the midpoint of each edge.

Figure 4 depicts statistically significant Spearman correlations among all variables included in the study. Associations included in the correlation network (Figure 4) were selected using a nominal Spearman *p* ≤ 0.015; notably, this threshold corresponded to an absolute correlation coefficient |ρ| ≥ 0.535 in all 59 selected pairs, indicating that the selected associations combined statistical significance with a substantial effect size. To assess the robustness of these associations to multiple testing, given the non-independence of several study variables (Methods, Section 4.12), we additionally computed cluster-stratified Benjamini–Hochberg FDR-corrected q-values (Supplementary Table S4). Thirty-six of the 59 originally selected pairs (61%) remained significant at FDR q ≤ 0.10, including the associations most central to our interpretation (e.g., vaccine dose number with metabolite PC1, TBARS, and carbonyl groups; CEF-specific cellular response with fever; and anosmia with ageusia). We consider the q ≤ 0.10 threshold appropriate in this context, as it has been proposed as a criterion for identifying potentially relevant associations in multi-omics integrative analyses [27]. Moreover, it retained several biologically expected relationships, such as the positive association between anti-Spike antibody titers and the number of vaccine doses, providing additional support for the biological plausibility of the selected network. Overall, in line with the hypothesis-generating philosophy underlying integrative multivariate analyses of biological data (see also [28] for a general discussion of this approach), the value of this network resides primarily in the coherence and biological plausibility of its emergent structure, rather than in the significance of any individual pairwise association—a criterion whose strict application would be statistically futile given the sheer number of possible comparisons relative to the sample size. This sensitivity analysis supports the overall robustness of the reported network, while a minority of associations—mainly those involving smaller sub-cohorts (e.g., granulocyte/monocyte and T-cell phenotyping subsets)—should be interpreted with additional caution given their reduced statistical power.

The network nodes represent individual characteristics, immune parameters, oxidative stress markers, metabolite and lipid profiles, and DNA damage indices; edges indicate significant correlations, with width and style reflecting increasing absolute Rho values, red denoting positive and blue negative associations.

A densely interconnected cluster is evident in the upper portion of the network. At its center, the number of vaccine doses emerges as a prominent hub, significantly associated with eight variables: it was positively linked to Omicron VOC infection and higher anti-Spike antibody levels, while being inversely associated with a longer interval between sampling and study cut-off, oxidative stress markers (carbonyl groups and TBARS), granulocyte PC2 (reflecting reduced monocyte–basophil activation), metabolite PC1 (associated with TCA-ketone linked amino acid metabolism), and rapid pulse symptom during infection.

Anti-Spike antibody levels were likewise inversely associated with granulocyte PC2 and carbonyl group intensity and were lower in individuals whose infection had occurred further from the sampling time point (PCO–ΔT). Carbonyl group levels were elevated in subjects who experienced a confusional state during infection—a symptom more frequently reported in Delta VOC cases—and increased with longer intervals between sampling and study cut-off date as well as between positivity and cut-off date (two intercorrelated variables). Carbonyl levels were also positively associated with both granulocyte PC2 and TBARS. TBARS levels were positively associated with rapid pulse, a symptom that was itself inversely correlated with vaccine dose number and co-occurred with shortness of breath, which was, in turn, associated with ibuprofen use and lipid PC2, the latter reflecting alterations in membrane-associated lipid metabolism. Metabolite PC1 showed a strong positive correlation with lipid PC1, which in turn was associated with concomitant therapy use during COVID-19. Notably, this treatment was predominantly reported by individuals with a history of previous pathology, a characteristic that was itself positively intercorrelated with allergies. Furthermore, these pre-existing conditions were inversely associated with ageusia, which was tightly linked to anosmia. Both symptoms were negatively correlated with T-cell PC4, representing the balance between DN terminally differentiated and memory DP T cells. Ageusia was also more frequent in individuals with a longer interval between vaccination and infection (VP–ΔT), a variable positively associated with metabolite PC3 (reflecting anaerobic glycolytic metabolism), which was in turn inversely correlated with %RD30 (closely related to %RD15).

The VP–ΔT interval was positively correlated with VS–ΔT, which was shorter in female subjects in this cohort. Women were also less frequently smokers and reported fever less often during COVID-19. Fever showed a strong inverse association with CEF-specific cellular response intensity and a negative association with Peptivator S responses, suggesting that robust pre-existing or rapidly inducible T cell competence may support viral control without triggering systemic febrile responses. Fever was also positively associated with anti-NP antibody titers. Peptivator S responses correlated with those to Peptivator N, which showed a weak positive association with *N*-acetylcysteine use during infection. Anti-NP antibodies were positively associated with myalgia, a symptom closely linked to arthralgia and frequently co-occurring with malaise/fatigue in individuals reporting a higher overall symptom burden.

Outside the main cluster (giant component—i.e., the largest set of mutually interrelated nodes), several smaller subnetworks were identified. These included a positive association between KL-9–specific cellular responses and intensive sports practice, a positive association between age and throat pain during infection, and a cluster linking LA-9–specific responses, T-cell PC2 (CD8-skewed terminal differentiation), and headache occurrence. All remaining variables are shown in the lower-left portion of Figure 4; none of them reached the predefined significance threshold for correlation with any other variable examined.

**Figure 5:**
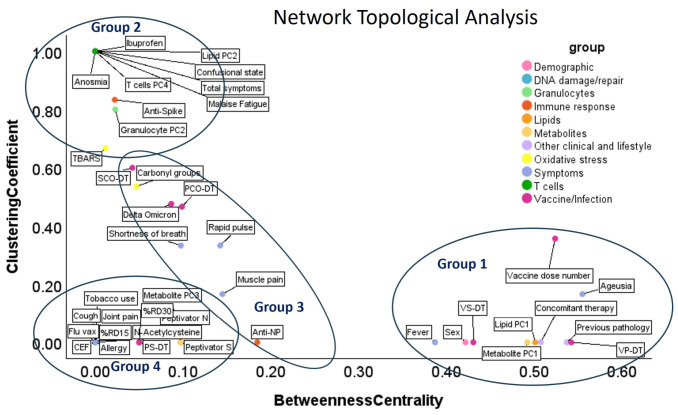
Network topological analysis. Scatter plot displaying all network nodes according to their betweenness centrality (x-axis) and clustering coefficient (y-axis). Four groups of variables (Groups 1–4) were identified based on their topological properties, as described in Section 2.9.

Network topology analysis using Cytoscape (Figure 5) characterized nodes by betweenness centrality—reflecting their role as bridges between network modules—and clustering coefficient, which measures local interconnectedness. Visual inspection of the scatter plot suggested four distinct groups of variables based on their topological properties. The first group comprised nodes with high betweenness and relatively low clustering, including vaccine dose number—which additionally showed the highest clustering coefficient within this group—followed by ageusia, lipid PC1, VP–ΔT, previous pathology, concomitant therapy, metabolite PC1, sex, fever, and VS–ΔT. The second group included variables with low betweenness but high clustering, indicating strong local submodule interconnections: total symptom number, T-cell PC4, anosmia, lipid PC2, confusional state, ibuprofen use, and malaise/fatigue, followed by anti-Spike antibodies, granulocyte PC2, and TBARS. The third group consisted of variables with both intermediate betweenness and clustering—SCO–ΔT, carbonyl groups, VOC classification, PCO–ΔT, shortness of breath, rapid pulse, muscle pain, and anti-NP antibodies—indicating limited integration within the global network structure. All remaining variables fell into a fourth group, characterized by low betweenness centrality and a low clustering coefficient, pointing to less relevant nodes for correlation fluxes within the interaction network.

Variables with high betweenness centrality may represent integrative nodes linking distinct biological and clinical dimensions, potentially reflecting factors that contribute to the coordination of immune, metabolic, and clinical features across the cohort. Conversely, variables with low betweenness but high clustering likely reinforce local interactions within defined submodules, reflecting tightly interconnected symptom clusters or related biological processes.

## 3. Discussion

The present work builds on and substantially extends our previous study [26], which identified novel immunogenic SARS-CoV-2 peptides and characterized the immune response in relation to individual characteristics. Here, we expanded both the study cohort and the analytical framework to explore potential associations among clinical, demographic and lifestyle features; humoral and cellular immune responses; peripheral T-cell and myeloid/monocytic phenotypes; systemic oxidative stress; DNA damage levels; and circulating metabolites and lipids in individuals recovered from mild COVID-19. To our knowledge, this represents one of the first attempts to integrate all these dimensions within a single study population.

In the present study, we focused on individuals recovering from mild COVID-19, excluding severe cases, in order to obtain a homogeneous study population with a low-noise biological background—a prerequisite for meaningful exploratory network analysis. Severe COVID-19, chronic inflammatory diseases, and major metabolic or cardiovascular comorbidities are known to profoundly affect immune function, oxidative stress, metabolism, and genomic stability independently of SARS-CoV-2 infection and would have introduced major confounding factors that could obscure the multidimensional relationships among biological domains that this study aimed to map. Restricting the analysis to mild convalescent subjects allowed these relationships to be explored under conditions of reduced clinical heterogeneity, providing a suitable and internally consistent framework for this exploratory systems-level analysis.

Overall, the analyzed population accurately reflects the local epidemiological scenario during the specified period, encompassing predominantly vaccinated individuals who were subsequently infected. Participants contracted SARS-CoV-2 during the Delta, Omicron BA.1 and Omicron BA.2 waves, capturing the real-world context in which vaccination became progressively available and was administered according to heterogeneous schedules. In this setting, not only the immune response to the viral Spike protein, but also the other observed features—such as immunological profiles, markers of oxidative stress, and DNA damage—likely reflect the combined effects of vaccination and infection.

From a translational perspective, the present work is consistent with its explicitly exploratory design. The multidimensional analytical strategy adopted here—technically complex and resource-intensive by nature—is not intended for routine clinical implementation, nor was it designed with that goal in mind. Rather, its value resides precisely in its capacity to simultaneously integrate immunological, metabolic, oxidative, and genomic information within the same individuals. Notably, all individual parameters fell within the physiological range typically observed in healthy adults—a finding that, taken in isolation, would have yielded little biological insight. It is only through their simultaneous integration that a structured network of biologically meaningful associations (both anticipated and novel) became detectable, illustrating the inherent limitations of conventional univariate approaches in capturing the coordinated complexity of host responses [28]. Systems-level analyses of this kind serve a specific and irreplaceable function in the research pipeline: they generate biologically coherent hypotheses, prioritize candidate pathways, and establish the integrative framework that will inform the design of more targeted, larger-scale prospective studies. Should the associations identified in this network be validated in larger cohorts or in studies designed ad hoc with sufficient statistical power, the resulting reduced sets of biological parameters could support clinically meaningful applications—including patient stratification based, e.g., on oxidative and metabolic profiles, the identification of subgroups that may benefit from targeted interventions such as antioxidant supplementation, and the development of personalized vaccination strategies informed by host-level immune and metabolic characteristics.

Among all variables examined, vaccine dose number occupied a central position within the correlation network, linking multiple clinical, immunological, oxidative, and metabolic variables. It combined high betweenness centrality—bridging distinct modules—with a relatively elevated clustering coefficient, indicating meaningful local interconnections. This configuration may underscore its role in linking infection variants, time intervals from infection and sampling, anti-Spike antibody levels, oxidative stress markers (carbonyl groups, TBARS), granulocyte activation (PC2), and metabolite PC1, highlighting its integrative relevance in the post-COVID-19 recovery landscape.

Other nodes with high betweenness centrality, including ageusia, lipid PC1, metabolite PC1, fever, and sex, similarly connected distinct clinical and biological domains. In contrast, variables characterized by lower betweenness but higher clustering—such as total symptom number, T-cell PC4, anosmia, lipid PC2, and malaise/fatigue—reinforced tightly interconnected local submodules, reflecting coherent interactions within symptom clusters or related immune–metabolic features. Together, these findings delineate a structured network architecture in which selected parameters function as integrative hubs, while others consolidate localized biological and clinical relationships across immune, metabolic, oxidative, and symptomatic dimensions. This integrative view reveals coordinated patterns across immune, metabolic, oxidative, and clinical parameters in individuals recovered from mild COVID-19.

Some of the observed correlations, such as the association between higher vaccine doses and elevated anti-Spike antibody levels, as well as expected patterns of symptom co-occurrence (fever, malaise/fatigue, anosmia/ageusia), are fully consistent with what has been reported in individuals recovered from COVID-19 with variable vaccination histories. Similarly, the correlation between smoking habit (captured by the tobacco use node) and male sex at birth confirms the national trend in the adult population. The recovery of several expected associations provides an internal consistency check for the analytical framework.

Interestingly, some associations with oxidative stress markers emerged from the network analysis. Both TBARS and carbonyl groups were negatively correlated with the number of vaccine doses, with carbonyl groups additionally showing a positive association with infections occurring further in the past. These findings are consistent with a possible association between vaccination and lower systemic oxidative stress markers in convalescent individuals, a relationship that has not yet been systematically investigated in the literature. However, given the cross-sectional study design and the lack of pre-vaccination baseline measurements, no causal or directional inference can be drawn, and reverse causation or residual confounding factors (e.g., differences in age, comorbidities, or time since infection across dose groups) cannot be excluded; confirmation in larger, controlled studies is warranted.

Different metabolomic studies suggest that COVID-19 vaccination induces systemic alterations in serum metabolites associated with shifts in TCA cycle intermediates, amino acids, and lipid species between high and low responders to mRNA vaccines [29] and between pre- and post-routine vaccination serum metabolomes [30]. Metabolite PC1, which explains the largest proportion of total variance and reflects overall energy-related metabolic signature encompassing amino acid turnover and tricarboxylic acid (TCA)-linked metabolism, was also negatively associated with vaccine dose number, suggesting that general metabolic function may be more perturbed in individuals who received fewer vaccine doses. Furthermore, it showed a strong positive correlation with lipid PC1, indicating a tight functional link between energy metabolism and lipid metabolism.

Granulocyte PC2, reflecting a reduced activation state of monocytes and basophils, was inversely correlated with vaccine dose number and positively associated with carbonyl group levels—a marker elevated in individuals with more remote infections. Together, these correlative associations are compatible with the hypothesis that lower vaccination coverage is linked to a residual immune imprint characterized by attenuated monocyte–basophil activation and heightened oxidative stress. This interpretation is consistent with evidence that vaccination induces durable epigenetic and functional reprogramming of circulating monocytes [31], conferring a long-lasting shift in innate immune responsiveness. Furthermore, basophils have been shown to adopt an activated phenotype during SARS-CoV-2 infection and may participate in shaping the humoral response, though their precise functional role in this context remains incompletely defined [32]. Finally, endogenous glutathione deficiency—well documented in COVID-19—has been proposed as a key driver of protein carbonylation and systemic oxidative stress, linking redox imbalance to both disease severity and impaired immune regulation [33]. As previously discussed with regard to other correlations, these associations should be interpreted cautiously, as the cross-sectional design of the study does not allow causal or directional inferences regarding the relationship between vaccine dose number, oxidative stress, granulocyte pattern and metabolite PC1 or granulocyte PC2.

Interestingly, the symptoms of ageusia and anosmia, which were strongly correlated with each other, were anticorrelated with T-cell PC4, a component characterized by high frequencies of both DN (double-negative) terminally differentiated T cells and EM (effector memory) DP (double-positive) T cells. In our cohort, these symptoms, observed primarily during Delta infections, are indicative of more pronounced disease manifestations even within otherwise mild COVID-19 cases [34]. The anticorrelation suggests that individuals experiencing loss of taste or smell have relatively lower frequencies of highly differentiated DN and EM DP T cells. The latter population has been characterized as a mature effector memory subset with antiviral functions [35], associated with advanced effector differentiation and rapid adaptive responses. This pattern may reflect a delayed or altered trajectory of T-cell maturation, possibly driven by differences in viral tropism, early immune priming, or local neuroinflammatory conditions during acute infection. Overall, the PC4 profile highlights a subgroup of convalescent individuals in whom ageusia and anosmia correspond to a distinct T-cell differentiation landscape, which could inform the understanding of symptom severity and immune response heterogeneity in mild COVID-19 [36].

An additional interesting observation emerges from the finding that fever during SARS-CoV-2 infection correlates with high titers of antibodies directed against the nucleocapsid (NP) protein, which is not included in Spike-based vaccines. This association suggests that febrile individuals may have undergone more intense viral replication and broader antigenic exposure, culminating in a more robust humoral response directed against internal viral proteins—a pattern consistent with the hypothesis that fever serves as a proxy for infection intensity rather than a mere symptom. Indirectly, this finding is consistent with the hypothesis that vaccination, by limiting viral replication and systemic inflammation, may attenuate the clinical manifestations of breakthrough infections, including febrile responses, as reported in previous studies [37].

Furthermore, we observed that subjects displaying a strong cellular response to the CEF peptide pool did not report fever during infection, indicating that pre-existing or rapidly inducible T-cell competence may contribute to effective viral control without triggering excessive systemic inflammatory responses. Notably, fever showed a negative association not only with CEF-specific responses but also with the overall Spike-specific T cell response (Peptivator S), further supporting the notion that broad T cell competence may contribute to attenuated febrile responses during infection. This is consistent with evidence showing that efficient T-cell immunity is associated with milder disease and improved viral clearance [38].

The weak positive association between the Peptivator N response and *N*-acetylcysteine use during infection may tentatively reflect the immunomodulatory and antioxidant properties of this compound, which has been shown to reduce oxidative stress and modulate Nuclear Factor kappa-light-chain-enhancer of activated B cells-(NF-κB)–dependent inflammatory pathways, potentially supporting T-cell functionality [39]. However, given the limited sample size and the observational nature of this finding, no causal inference can be drawn.

The observation that individuals with high anti-NP antibody titers also frequently reported myalgia, which in turn correlated with arthralgia and with the overall number of symptoms, suggests that a broader systemic inflammatory response accompanied natural infection in this subgroup. Musculoskeletal symptoms such as myalgia and arthralgia are well-recognized manifestations of cytokine-driven inflammation during acute SARS-CoV-2 infection and have been associated with increased innate immune activation and higher inflammatory mediator levels [40]. Moreover, several studies have shown that greater clinical symptom burden correlates with stronger humoral responses, particularly against internal viral proteins such as nucleocapsid, likely reflecting higher viral replication and antigen exposure [41,42]. Together, these findings support the interpretation that anti-NP titers may act as a surrogate marker of infection intensity, characterized by systemic inflammation and multi-symptom involvement.

The positive correlation between time from vaccination to breakthrough infection and metabolite PC3 suggests that the metabolic profile captured by this component, characterized mainly by lactate and alanine, varies according to the post-vaccination interval. The inverse association between PC3 and %RD30 indicates that this metabolic configuration may be linked to cellular adaptive responses that limit the persistence of DNA damage after repair incubation. Although longer intervals from vaccination are generally associated with waning immune protection, the observed associations are more likely to reflect host metabolic adaptations to infection-related stress than direct correlates of vaccine-induced immunity. This interpretation is consistent with evidence that metabolic reprogramming and oxidative stress responses play an important role in determining cellular resilience during SARS-CoV-2 infection [43]. Our data are consistent with the notion that vaccine-induced immune training shapes the metabolic landscape during subsequent infection, potentially influencing cellular stress responses. Overall, these observations highlight a dynamic interplay between vaccination timing, immunometabolic programming, and genomic stress, suggesting that metabolic signatures such as PC3 may reflect adaptive responses or compensatory mechanisms rather than a simple correlate of protection. Longitudinal studies integrating metabolomics, redox markers, and functional immune assays will be crucial to clarify causality and determine the biological significance of these patterns.

Finally, we observed that the cellular response to two individual Spike-derived peptides was significantly associated with specific clinical and phenotypic features. In particular, T-cell reactivity to the LA-9 peptide correlated with the occurrence of headaches during SARS-CoV-2 infection and with PC2 of the T-cell compartment, characterized by a CD8-skewed terminal differentiation profile. Similarly, the response to the KL-9 peptide was associated with intense physical activity, as already reported in our previous study. These findings suggest that epitope-specific cellular responses are not uniformly distributed across individuals but may instead reflect distinct immunological and host-related contexts. The association between LA-9–specific responses and headache during infection is intriguing. Headache is a common neurological manifestation of COVID-19 and has been linked to systemic inflammation, cytokine release, and neuroimmune activation. A CD8-skewed, terminally differentiated T-cell profile—captured by PC2—may reflect a cytotoxic, highly differentiated effector phenotype characterized by increased expression of granzyme/perforin and reduced proliferative capacity. Such a profile has been described in the context of strong antigenic stimulation and viral clearance but also in association with heightened inflammatory responses. It is therefore plausible that individuals mounting a robust, epitope-specific CD8 response to LA-9 may have experienced a more pronounced inflammatory milieu, contributing to neuroinflammatory symptoms such as headache. The link between KL-9–specific responses and intense physical activity is also biologically plausible. Regular and vigorous exercise is known to modulate T-cell homeostasis, promoting enhanced immune surveillance, improved mitochondrial fitness, and functional responsiveness of cytotoxic T lymphocytes [44]. More broadly, these data reinforce the concept that SARS-CoV-2-specific cellular immunity is shaped not only by viral and vaccine-related factors but also by host characteristics, including immunophenotypic architecture and lifestyle variables.

We would like to emphasize that the overall cohort size (*n* = 20) is small relative to the number of variables and comparisons examined and that several sub-analyses were performed on even smaller subsets (*n* = 18 for T-cell phenotyping, *n* = 15 for HLA-A*02:01-restricted epitope-specific cellular responses, *n* = 13 for granulocyte phenotyping, and *n* = 10 for DNA damage analysis). All associations reported—including those emerging from PCA and network analyses—should therefore be regarded as exploratory and hypothesis-generating, consistent with the study design, and requiring validation in larger, adequately powered cohorts before any biological or clinical inference can be drawn. Given the sample size, no multivariable adjustment for vaccine platform, dose number, infecting variant, or time intervals was performed; instead, these variables were included directly as nodes in the correlation network, allowing their associations with other study parameters to be examined explicitly rather than statistically controlled for. This approach cannot fully disentangle the independent contribution of each factor, and residual confounding among correlated exposures (e.g., dose number and time since vaccination) cannot be excluded. This relatively modest sample size warrants caution in the interpretation of some observed correlations, which may partly reflect confounding rather than direct biological relationships. For example, the apparent association between sex and the interval from vaccination to sampling is likely driven by cohort size, whereas the inverse correlation between sex and fever during COVID-19 aligns with previously reported sex-based differences in symptom presentation. Clinical studies indicate that males are more likely than females to develop fever during SARS-CoV-2 infection, reflecting broader sex-based differences in immune responses. Men tend to exhibit stronger inflammatory responses and higher disease severity, while females often mount more robust innate and adaptive immunity. These differences are thought to involve sex hormones, X-linked immune genes, and hormone-mediated immune modulation [45].

The present study has several additional notable limitations that should be considered when interpreting the findings, beyond the small sample size already discussed above. By design, this study aimed to characterize patterns and relationships across multiple biological dimensions rather than to establish causality, and the observed correlations should therefore be interpreted accordingly, as exploratory and hypothesis-generating rather than as confirmatory or definitive findings. Because the cohort was intentionally restricted to individuals with a paucisymptomatic or mild course of COVID-19, the findings reported here cannot be extrapolated to individuals with moderate-to-severe disease, to hospitalized patients, or to those with long COVID, in whom immune, metabolic, and oxidative stress profiles are known to differ substantially.

Key structural limitations of this study include the absence of healthy control subjects, disease-control cohorts such as individuals recovering from other respiratory viral infections, and baseline pre-infection samples with longitudinal follow-up. These constraints prevent assessment of whether the observed signatures are specific to SARS-CoV-2 or reflect more general antiviral host responses and limit the possibility of evaluating temporal dynamics and causal relationships among the investigated variables. The relatively small number of unvaccinated participants further prevents a robust disentanglement of infection-related from vaccination-related effects. Moreover, causal inference remained largely unclear: for instance, it is not possible to determine whether *N*-acetylcysteine directly enhances antigen-specific cellular responses or whether individuals with stronger immune activation were independently more inclined to use the compound during infection. Taken together, these constraints confirm that the associations reported here should be interpreted as exploratory relationships within this specific recovered cohort rather than as COVID-19-specific biological signatures. Symptom data were collected retrospectively through self-report at a single interview conducted at the time of enrollment and are therefore subject to recall bias, which may be differential with respect to time since infection or symptom severity. In addition, since participation required voluntary enrollment and provision of informed consent, a degree of selection bias toward more health-engaged or symptom-aware individuals cannot be excluded.

Finally, the heterogeneity of the cohort—encompassing different SARS-CoV-2 variants, vaccination schedules, and time intervals from infection—while reflective of real-world conditions, introduces additional variability that may influence the observed associations and complicate direct comparisons across subgroups.

The present work raises several questions that warrant dedicated follow-up investigation. Future studies should validate these findings in larger prospective cohorts, including healthy individuals, patients with other acute respiratory viral infections, and longitudinal sampling before infection, during the acute phase, and throughout recovery. Such study designs will be essential to distinguish SARS-CoV-2-specific biological signatures from general antiviral host responses and to evaluate the reproducibility and generalizability of the network architecture identified in the present work. Extending this integrative approach to severe COVID-19 and long COVID cohorts represents a natural and compelling next step for future investigation. In individuals with long COVID, in whom persistent immune dysregulation, oxidative stress, and metabolic perturbations have been reported, this multidisciplinary framework could provide a valuable comparative perspective and help identify biological signatures that distinguish full recovery from prolonged post-infectious sequelae [3,13,21].

Among the most compelling are the potential role of vaccination in modulating systemic oxidative stress and its downstream effects on metabolic and myeloid compartments—observations that, to our knowledge, have received limited attention in the post-COVID-19 literature and deserve further validation in larger, prospectively designed cohorts.

The relationship between anaerobic glycolytic metabolism and vaccine-infection interval suggests a previously underappreciated link between immunometabolic programming and genomic integrity in the context of breakthrough infection. Elucidating the directionality and mechanistic basis of this relationship will require longitudinal studies incorporating metabolomics, redox profiling, and functional immune assays at multiple time points relative to vaccination and infection.

The association between epitope-specific T cell responses and host-level variables—such as lifestyle factors and symptom profiles—further highlights the need to move beyond aggregate immune measures toward a more granular, individual-level characterization of cellular immunity. Whether these epitope-specific signatures have prognostic value or functional relevance in protection against reinfection or severe disease remains an open question.

## 4. Materials and Methods

### 4.1. Study Workflow

The overall study workflow is illustrated in Figure 1. Participants were screened and enrolled according to predefined criteria (see Section 4.2). Demographic, clinical, and lifestyle data, including infection and vaccination history and symptoms experienced during COVID-19, were collected through a structured survey questionnaire administered on the day of blood collection.

Following blood sampling, an aliquot of peripheral blood was processed to isolate and cryopreserve peripheral blood mononuclear cells (PBMCs) and plasma. A separate aliquot of fresh whole blood was used for multiparametric flow cytometry (MFC) immunophenotyping of naïve/memory T cell subsets and granulocyte/monocyte populations, as well as for the assessment of DNA damage and repair capacity.

Thawed cryopreserved PBMCs (cPBMCs) were used in IFN-γELISpot assays to evaluate SARS-CoV-2–specific T cell responses. Plasma samples were analyzed for anti-Spike and anti-nucleoprotein (NP) antibodies by electrochemiluminescence immunoassay and for oxidative stress markers (TBARS and protein carbonyl groups) by spectrophotometric assays and subjected to comprehensive metabolomic and lipidomic profiling by nuclear magnetic resonance (NMR) spectroscopy.

To facilitate data integration, selected groups of variables—T cell naïve/memory phenotypes, granulocyte/monocyte phenotypes, metabolomic profiles, and lipidomic profiles—were subjected to Principal Component Analysis (PCA). The resulting principal components (PCs), together with raw variables not subjected to dimensionality reduction (e.g., oxidative stress markers, antibody responses, DNA damage indices, and individual characteristics), were included in Spearman correlation analysis. Significant correlations were visualized by means of a network-based representation, providing an integrative overview of immune, metabolic, and clinical responses following infection and vaccination.

### 4.2. Subjects

#### 4.2.1. Inclusion Criteria

Subjects were eligible for enrollment if they met all of the following criteria: age between 18 and 70 years; laboratory-confirmed SARS-CoV-2 infection and subsequent negativization (both determined by PCR or rapid antigen test); negativization occurring 30 to 180 days before the enrollment date; previous asymptomatic or mild COVID-19 course; good general health conditions; ability to understand and willingness to comply with planned study procedures; and provision of written informed consent.

#### 4.2.2. Exclusion Criteria

Subjects were excluded if any of the following conditions applied: previous severe COVID-19 (pneumonia or hospitalization); concurrent metabolic diseases (including obesity, diabetes, resistant hypertension, severe cardiovascular disease, malignancies, or rheumatic disease); chronic infectious disease (HIV, HBV, or HCV); concomitant biological, antibiotic, or immunosuppressive therapy; use of immunosuppressive drugs during COVID-19; inability to provide informed consent; or withdrawal of previously signed informed consent.

#### 4.2.3. Demographic, Clinical, and Lifestyle Data Collection

On the day of blood collection, participants were interviewed to obtain information on their SARS-CoV-2 vaccination and infection history, as well as their past and current health status. Key demographic, vaccination, and infection data are summarized in Table 1; reported symptoms during COVID-19 are listed in Supplementary Table S1; and additional clinical and lifestyle characteristics are reported in Supplementary Table S2. The likely infecting SARS-CoV-2 VOC was estimated based on the date of the first positive swab, by reference to the COVID-19 Data Portal (CDP; https://www.covid19dataportal.org/), an open-access data-sharing resource, accessed on 18 December 2022 [46].

### 4.3. Blood Sampling

Blood samples were collected by trained personnel at S. Filippo Neri Hospital (ASL RM1, Rome, Italy). A total of 30 mL of venous blood was drawn into lithium-heparin Vacutainer tubes (Becton Dickinson, Franklin Lakes, NJ, USA) and processed within 2 h of collection. An aliquot of fresh whole blood was committed to HLA typing and immunophenotyping. Plasma was obtained by centrifugation and immediately stored at −80 °C. PBMCs were isolated by Ficoll density gradient centrifugation (Lymphoprep, Axis-Shield PoC, Oslo, Norway) and cryopreserved in liquid nitrogen until use, as previously described [47].

### 4.4. Anti-Spike and Anti-NP Antibody Quantification

Plasma samples were tested for anti-SARS-CoV-2 anti-NP using the Elecsys^®^ Anti-SARS-CoV-2 electrochemiluminescence immunoassay (Roche Diagnostics, Basel, Switzerland) on a cobas e411 analyzer. This double-antigen sandwich assay uses recombinant nucleocapsid protein (NP) for the detection of total antibodies (IgA, IgM, and IgG). Results are expressed as a cut-off index (COI = signal/cut-off); values ≥ 1.0 were considered reactive (positive).

Anti-SARS-CoV-2 Spike receptor-binding domain antibodies (anti-Spike) were quantified using the Elecsys^®^ Anti-SARS-CoV-2 S assay (Roche Diagnostics International Ltd, Rotkreuz, Switzerland), which provides quantitative detection of total antibodies against the Spike receptor binding domain (RBD). Results are expressed in U/mL, traceable to the assay’s proprietary internal standard (Roche Diagnostics), comprising an equimolar mixture of two monoclonal antibodies binding Spike-1 RBD at distinct epitopes (1 nM = 20 U/mL) [48]. The assay cut-off is 0.8 U/mL, with a linear range up to 250 U/mL; samples exceeding this threshold were diluted up to 1:1000 in specimen diluent before re-analysis. Individual anti-NP and anti-Spike antibody titers are shown in Supplementary Figure S1.

### 4.5. HLA-A*02 Typing

HLA-A*02 positivity was determined by staining 50 µL of fresh whole blood with a Fluorescein Isothiocyanate (FITC)-conjugated anti-human HLA-A*02 antibody (clone BB7.2, BioLegend, San Diego, CA, USA), and results are reported in Table 1.

### 4.6. IFN-γ ELISpot Assay

The IFN-γ ELISpot assay was performed as previously described [26], using pre-coated 96-well plates with nitrocellulose membranes (Merck-Millipore, Burlington, MA, USA). Briefly, cPBMCs were thawed and cultured in complete medium (ThermoFisher Scientific/Gibco: Waltham, MA, USA) under standard conditions (37 °C, 5% CO_2_) and stimulated for 24 h, followed by spot development according to the manufacturer’s instructions (Mabtech, Nacka Strand, Sweden).

All subjects’ cPBMCs were stimulated with Peptivator^®^ S and Peptivator^®^ N overlapping peptide pools (Miltenyi Biotec, Bergisch Gladbach, Germany; 10 µg/mL). Staphylococcal enterotoxin B (SEB; Sigma-Aldrich, Munich, Germany; 2 µg/mL) served as a positive control and the custom-synthesized VF-9 peptide (10 µg/mL) as a negative control. For the 15 HLA-A*02:01–positive subjects, additional stimulations were performed with the custom-synthesized Spike-derived peptides LA-9 and KL-9, as well as the CEF positive control peptide pool (10 µg/mL each). In all conditions, the co-stimulatory anti-CD28 antibody (BD Biosciences, Franklin Lakes, NJ, USA) was added at 1 µg/mL.

Results are expressed as the ratio of SFC counts induced by each peptide stimulus to those induced by the irrelevant VF-9 negative control peptide (SFC Index, SFCI). The VF-9 peptide consistently generated equal or fewer spots than medium alone, anti-CD28, or dimethyl sulfoxide (DMSO)-only controls.

### 4.7. Synthesis of HLA-A*02:01–Restricted Peptides

The Spike-derived peptides LLFNKVTLA (LA-9) and KIADYNYKL (KL-9), the irrelevant control peptide VTWFHAIHF (VF-9), and the CEF positive control peptide pool were synthesized by BioFabResearch (Rome, Italy) at >95% purity (endotoxin-free) and provided in lyophilized form. Peptides were reconstituted in DMSO at 40 mg/mL and used at a final culture concentration of 10 µg/mL, avoiding repeated freeze–thaw cycles. The CEF pool was designed as an equimolar mixture of three HLA-A*02:01–restricted immunodominant epitopes: Cytomegalovirus (CMV) pp65 NLVPMVATV, Epstein–Barr virus (EBV) LMP2 CLGGLLTMV, and Influenza virus (FLU) M1 GILGFVFTL.

### 4.8. Multiparametric Flow Cytometry Immunophenotyping

#### 4.8.1. Naïve/Memory T Cell Subset Characterization in Peripheral Blood

Peripheral blood T cell naïve/memory status was assessed by staining fresh whole blood with a 7-color panel of fluorochrome-conjugated antibodies (Supplementary Table S3a), based on DuraClone technology (Beckman Coulter, Life Sciences, Geneva, Switzerland). The panel comprised five antibodies in dry formulation (anti-CD3, -CD4, -CD8, -CD45RA, -CCR7) combined with two antibodies added in liquid form (anti-CD45, Beckman Coulter, Brea, CA, USA; anti-Vδ2, BioLegend, San Diego, CA, USA). DuraClone reagents have demonstrated high reproducibility and standardization capacity in large-scale immunophenotyping projects [49,50].

Within the CD3^+^ T cell compartment, the following subsets were defined: CD4 single-positive (CD4sp), CD8 single-positive (CD8sp), double-positive CD8^hi^CD4^low^ (DP1), double-positive CD8^low^CD4^hi^ (DP2), double-negative CD4^−^CD8^−^ (DN), and Vδ2^+^ γδ T cells. Within each subset, maturation status was determined based on CD45RA and CCR7 co-expression, defining naïve (N; CD45RA^+^CCR7^+^), central memory (CM; CD45RA^−^CCR7^+^), effector memory (EM; CD45RA^−^CCR7^−^), and terminally differentiated (TD; CD45RA^+^CCR7^−^) phenotypes. The gating strategy is illustrated in Supplementary Figure S2; the distribution of defined subpopulations is shown in Supplementary Figure S3.

#### 4.8.2. Peripheral Blood Granulocyte and Monocyte Characterization

Granulocyte and monocyte subsets were characterized in fresh peripheral blood using 10-color Granulocyte DuraClone™ tubes (Beckman Coulter, Marseille, France, Supplementary Table S3b). The gating strategy and representative dot plots are shown in Supplementary Figure S4. Briefly, leukocytes were identified based on CD45 expression and side scatter, following exclusion of debris, doublets (singlet discrimination), and events with unstable acquisition kinetics (time gate).

Granulocyte subsets were gated as leukocyte cells negative for lineage markers (CD3, CD14, CD56, and CD19) to exclude lymphoid and monocytic populations. Within this gate, neutrophils were identified as CD294^−^CD15^+^CD16^+^ cells, eosinophils as CD294^+^CD15^+^CD16^int^ cells, and basophils as CD294^+^CD15^−^CD16^−^ cells. Monocytes were identified as CD33^+^ lineage-intermediate cells and further subdivided into: classical monocytes (CD16^−^), representing the predominant phagocytic subset; non-classical monocytes (CD16^+^), involved in vascular patrolling and antiviral responses; and Gr–Mo doublets, characterized by a granulocyte-like phenotype within the lineage-negative gate, high CD16 expression, and reduced monocytic features, consistent with a distinct inflammatory and regulatory profile.

All granulocyte and monocyte subsets were further analyzed for surface expression of CD11b (activation marker), CD62L (L-selectin, an adhesion molecule downregulated upon cell activation), and PD-L1/CD274 (immune checkpoint molecule upregulated in inflammatory contexts), reported as MFI. Frequencies of all subsets were calculated relative to their corresponding parent population, with eosinophils and basophils additionally expressed as percentages of the CD294^+^ compartment. Supplementary Figure S5 presents the distribution of all subsets as percentages of the parent population, together with corresponding MFI values for each activation marker.

#### 4.8.3. Flow Cytometry Acquisition and Analysis

Data were acquired on a Gallios flow cytometer (Beckman Coulter, CA, USA) and analyzed using Kaluza software v.1.3 (Beckman Coulter, Brea, CA, USA). A minimum of 200,000 events in the leukocyte gate were acquired per sample.

### 4.9. Metabolomic and Lipidomic Profiling

NMR-based metabolomic analysis was performed on intact plasma samples, which were diluted to a final volume of 0.7 mL with 0.9% NaCl prepared in D_2_O, and analyzed using a Bruker AVANCE NEO 600 spectrometer (14.1 T; Bruker BioSpin, Karlsruhe, Germany) [51]. For each plasma sample, three complementary molecular-window approaches were employed: (a) a standard one-dimensional nuclear Overhauser effect spectroscopy pulse sequence (1D NOESY-presat) to detect signals from both low- and high-molecular-weight compounds; (b) a standard one-dimensional spin-echo Carr–Purcell–Meiboom–Gill sequence (1D CPMG) optimized for the detection of aqueous low-molecular-weight metabolites; and (c) a standard diffusion-edited pulse sequence primarily designed to highlight macromolecular signals, particularly lipoproteins. Spectral processing, including phase and baseline correction, was carried out using Bruker TopSpin version 4.1.4. Metabolite levels were expressed as percentages relative to the total pool of investigated metabolites (% of total metabolites/lipids).

### 4.10. Oxidative Stress Assessment

TBARS quantification was performed according to [52]. Plasma aliquots were mixed with TBA reagent and incubated under acidic conditions at 95 °C for 60 min to allow the formation of an adduct between TBA and malondialdehyde (MDA). After cooling, samples were centrifuged to remove precipitates, and the absorbance of the supernatant was measured spectrophotometrically at 532 nm. TBARS concentration was based on the molar extinction coefficient of MDA (1.56 M^−1^ cm^−1^).

Protein carbonyl groups were measured according to the method by Reznick and Packer, slightly modified [53]. Briefly, blood plasma was brought to react with 0.1% (*v*/*v*) freshly prepared from a 10% stock in 50 mM Hepes buffer, pH 7.2. Samples were incubated for 15 min at room temperature and centrifuged at 12,000 rpm for 10 min. Fifty μl of the resulting supernatants were brought to react with 200 μL of 2,4-dinitrophenylhydrazine (DNPH; 10 mM in 2.5M HCl). Blanks received HCl without DNPH. Samples were incubated for 1h at room temperature in the dark, with intermittent vortexing every 15 min. Samples were then treated with 250 μL of 20% (*w*/*v*) Trichloroacetic Acid (TCA), vigorously vortexed, incubated for 10 min on ice, and centrifuged for 5 min at 15,000 rpm. Supernatants were discharged, and 200 μL of 10% TCA was added to pellets. Samples were vigorously vortexed, incubated for 10 min on ice, and centrifuged for 5 min at 15,000 rpm. Supernatants were discarded. The resulting pellets were washed three times with 200 μL of ethanol/ethyl acetate (1/1 *v*/*v*) incubating each time for 10 min before centrifuging for 5 min at 15,000 rpm. After the third wash, pellets were resuspended in 100 μL of 6 M guanidine in 20 mM potassium phosphate adjusted at pH 2.3 with TCA. After 10 min at 37 °C, samples were centrifuged for 5 min at 10,000 rpm. Supernatant absorbance was measured in a 96-well plate reader (Victor NIVO, Perkin Elmer). After subtracting the value of the own blank from each sample, protein carbonyl content was calculated by using the molar absorption coefficient of 22,000 M^−1^ cm^−1^. Both TBARS and protein carbonyls were expressed in nmol/mg protein.

### 4.11. Analysis of DNA Damage and Repair Kinetics

Basal and radiation-induced DNA damage were assessed using the alkaline comet assay before and immediately after treatment, while residual DNA damage was evaluated after 15 and 30 min of incubation at 37 °C. Samples were processed according to the alkaline comet assay protocol described in detail elsewhere [54]. Briefly, cells were suspended in 0.7% (*w*/*v*) low-melting point agarose (Bio-Rad Laboratories, Hercules, CA, USA) and distributed onto a lower layer of 1% (*w*/*v*) normal-melting point agarose (Bio-Rad Laboratories, Hercules, CA, USA) on microscope slides. The slides were immersed in a lysis solution (2.5 M NaCl, 100 mM Na2 EDTA and 10 mM Tris, pH 10) containing 10% DMSO and 1% Triton X-100 and incubated overnight at 4 °C. After lysis, slides were placed in a horizontal electrophoresis tank with alkaline electrophoresis buffer (300 mM NaOH and 1 mM Na2 EDTA, pH 13) and left in the solution for 25 min at 4 °C to allow the DNA to unwind and the alkali-labile sites to express. Electrophoresis was carried out at 4 °C for 20 min, 20 V (1 V/cm), 300 mA. After electrophoresis, slides were immersed in 0.3 M sodium acetate in ethanol for 30 min. Slides were dehydrated in absolute ethanol at −20 °C for 10 min and air-dried at room temperature. Immediately before scoring, slides were stained with 12 µg/mL ethidium bromide and analyzed with a fluorescence microscope (Leitz, Wetzlar, Germany). One hundred cells were scored for each experimental point from two slides. DNA damage was quantified by the percentage of DNA in the tail, indicated as Tail Intensity (%TI), calculated using an Image Analysis Software V3. (IAS, Delta Sistemi, Rome, Italy). Nowadays, %DNA is the parameter recommended to measure DNA damage in the comet assay since it is most linearly related to the amount of single-strand breaks and the easiest to intuitively understand. For the analysis of DNA repair kinetics, the percentage of residual DNA damage at different time points, 15 and 30 min (t), after irradiation (%RD DNA) was calculated as follows: (DNA damage at time t after irradiation − DNA damage in control cells before irradiation)/(DNA damage immediately after irradiation − DNA damage in control cells before irradiation) × 100.

### 4.12. Statistical Analysis

Laboratory analyses were conducted on coded, pseudonymized samples by personnel blinded to participants’ vaccination history and clinical characteristics. Clinical and demographic data were linked to the laboratory dataset only after completion of the laboratory analyses and generation of the raw analytical results. All study parameters were entered into an SPSS database (IBM SPSS Statistics v.30, IBM Corp., New York, NY, USA), including demographic, vaccination, and infection-related data (Table 1); symptoms (Supplementary Table S1); clinical and lifestyle characteristics (Supplementary Table S2); immune response variables (Supplementary Figure S1); T lymphocyte subpopulations (Supplementary Figure S3); granulocyte and monocyte subpopulations (Supplementary Figure S5); metabolomic and lipidomic data (Supplementary Figure S6); as well as oxidative stress and DNA damage/repair variables (Supplementary Figure S7).

Principal Component Analysis (PCA) was applied separately to four groups of variables—T cell naïve/memory phenotypes, granulocyte/monocyte phenotypes, metabolomic profiles, and lipidomic profiles—to reduce dimensionality and identify major patterns of interindividual variation, using the built-in PCA module in SPSS. For T cell and granulocyte/monocyte datasets, given the limited number of subjects relative to the number of parameters, PCA was performed on a transposed dataset in which variables were treated as cases and subjects as variables, thus exchanging the role of component loadings and scores. This approach enhances the interpretability of PCA in high-dimensional, low-sample-size settings, as previously described [55]. All data were normalized and scaled prior to PCA.

The relative contribution of each immune cell subset to the identified principal components was calculated from component scores and visualized using pie charts (Figure 2), providing an intuitive representation of the variables driving each component. For metabolomic and lipidomic PCs, variable contributions are reported as loading coefficients and visualized as heatmaps (Figure 3), enabling a clear visualization of the metabolite and lipid species shaping each component. Each PC was assigned a label reflecting the dominant biological theme captured by its leading variables.

Specific time intervals were calculated in days: PS-ΔT, VS-ΔT, VP-ΔT, PN-ΔT, PCO-ΔT and SCO-ΔT (see Section 2.1).

Non-parametric Spearman’s rank correlation analysis was used to assess associations between all pairs of variables under investigation, including extracted PCs—treated as continuous variables and replacing the original parameters from which they were derived—raw immune response parameters, oxidative stress markers, DNA damage parameters, and individual characteristics. Correlations involving dummy variables were excluded when fewer than three observations were present in any category, to avoid unreliable estimates. Nominal variables with one or more categories containing fewer than three observations were similarly excluded from comparative analyses (e.g., vaccine manufacturer subgrouping). An aggregated binary VOC variable (Delta vs. Omicron, combining Omicron BA.1 and Omicron BA.2) was generated to explore differences between these two major variant groups.

Based on the resulting correlation matrix, a network was constructed (Figure 4).

Given the exploratory, hypothesis-generating nature of this study—aimed at detecting a coherent structure of association across biologically interconnected domains rather than testing a set of independent, pre-specified hypotheses [28]—associations for the correlation network (Figure 4) were selected using a nominal Spearman *p* ≤ 0.015, corresponding in this dataset to |ρ| > 0.535 for every selected pair, thus combining statistical significance with a substantial effect size. This threshold was empirically chosen as the least stringent cut-off at which the network formed a single connected giant component encompassing the majority of study variables, allowing the topological analyses of betweenness centrality and clustering coefficient (Figure 5) to retain biological meaning; stricter thresholds fragmented the network, while more permissive ones added edges without a proportional gain in connectivity. We acknowledge that this choice was made to preserve analytical tractability rather than to control the false discovery rate, and all resulting associations should be interpreted as exploratory. In this respect, it is worth noting that an empirical threshold for setting links between nodes of a graph is a usual practice in network science (see [56]).

To verify the robustness of this approach, a sensitivity analysis based on Benjamini–Hochberg FDR correction was performed and is reported in Supplementary Table S4. Because the 61 study variables were organized a priori into biologically distinct domains and included several non-independent measures (e.g., shared-derived time intervals, repeated measurements of the same biological process, and clinically related symptom clusters), correction was applied within these predefined families rather than across the entire set of pairwise comparisons (m = 1830), consistent with the framework of family-wise and hierarchical multiple testing, in which FDR is controlled within conceptually related hypothesis groups rather than across heterogeneous ones [57]. Variables were grouped into 13 pre-specified clusters (demographic, exposure/vaccination, time intervals, symptoms, clinical history/lifestyle, antibody response, cellular response, T-cell principal components, granulocyte/monocyte principal components, oxidative stress, DNA damage, metabolite principal components, and lipid principal components), and Benjamini–Hochberg correction was applied separately within each cluster-pair family. Raw and cluster-adjusted q-values for all tested pairs, together with variable cluster assignments, are provided in Supplementary Table S4. A threshold of q ≤ 0.10 was considered in this context, as this value has been proposed as a criterion for identifying potentially relevant associations in multi-omics integrative analyses [27].

Network visualization and topology analysis were performed using Cytoscape v.3.10.3 [58]. Clustering coefficient and betweenness centrality were extracted for each node using the software’s built-in network analysis tool and displayed in a scatter plot (Figure 5) to characterize the structural role of individual variables. Clustering coefficient values (clustering coefficient corresponds to the relative frequency of ‘complete triads’, which holds the condition: if variable A is correlated with variable B and variable B is correlated with variable C, then even variable A is directly correlated with variable C. While the clustering coefficient has to do with the local network structure, with high clustering coefficient nodes being the nuclei of small clusters of interrelated variables, betweenness deals with the long-range signal transmission throughout the network. Betweenness of a node corresponds to the number of ‘shortest paths’ traversing it; in other words, a “high betweenness” node corresponds to a node (variable) involved in many signal transmission pathways across the network [59]. Fragmented subnetworks consisting of two or three nodes were excluded from the topological analysis, and only nodes belonging to the giant component of the network were considered. Based on their distribution across betweenness centrality and clustering coefficient, nodes were grouped into four categories by visual inspection of the resulting scatter plot; group boundaries were defined qualitatively and do not correspond to a formal statistical clustering procedure.

## 5. Conclusions

Our integrative analysis in individuals recovered from mild COVID-19 reveals a structured and biologically coherent network of associations spanning subjects’ characteristics, vaccination history, immune responses, oxidative stress, metabolite and lipid profiles, and DNA integrity. Higher numbers of vaccine doses were associated with elevated anti-Spike antibody levels, lower oxidative stress markers, and more balanced metabolic and myeloid/monocytic profiles; these correlative findings are compatible with, but do not establish, a potential beneficial role of vaccination extending beyond antibody induction and warrant further investigation in prospective, longitudinal studies.

Symptom-specific patterns—including the association of anosmia/ageusia with a distinct T cell differentiation landscape, as well as the link between fever, anti-NP humoral responses, and a reduced cellular response—suggest that even mild COVID-19 clinical manifestations reflect underlying immunometabolic and host-dependent heterogeneity.

Collectively, these findings underscore the multifaceted impact of vaccination and infection on immune, metabolic, and genomic parameters and provide an observational framework for understanding host responses to mild SARS-CoV-2 infection. Although the specific findings reported here require validation in larger longitudinal cohorts, this work illustrates how a multidisciplinary approach—combining clinical, immunological, metabolic, and genomic dimensions—can capture the complex constellation of biological processes activated during infectious disease, offering a template for similarly comprehensive investigations across diverse infectious or inflammatory contexts, and represents a widely applicable systems biology framework that may help identify coordinated biological signatures underlying host response variability. Beyond the specific context of COVID-19, the multidimensional profiling strategy adopted here may hold broader significance for the field more generally. In particular, this approach may contribute to improving our understanding of host–pathogen interactions; facilitating the identification of biological endotypes associated with immune resilience or susceptibility; supporting the development of personalized vaccination and immunomodulatory strategies; and providing systems-level frameworks that can be adapted to future emerging infectious diseases and pandemic preparedness.

## Figures and Tables

**Table 1:** Subjects’ main characteristics.

Subj# ID	1st Positive Swab Date	Sex at Birth	Probable VOC	Vaccine Dose Number	Vaccine Manufacturer ^#^	Age	Total Symptom Number	PS-ΔT	VS-ΔT	VP-ΔT	PN-ΔT	PCO-ΔT	SCO-ΔT	
	(d-m-y)					(Years)		(Days) ^§^						
01 *	23.07.2021	M	B 1.617.2 Delta	1	P	18	4	165	169	4	15	357	192	
02 *	26.07.2021	F		1	M	31	1	116	147	31	9	354	238	
03	21.08.2021	M		0	NA	41	5	202	NA	NA	34	328	126	
04 *	11.09.2021	F		1	J	51	7	69	170	101	10	307	238	
05 *	19.10.2021	F		1	J	55	13	52	206	154	26	269	217	
06 *	05.11.2021	M		3	MMP	41	5	173	434	261	16	252	79	
07 *	28.12.2021	F	B.1.1.529 Omicron BA.1	3	PPP	44	4	76	90	14	22	199	123	
08 *	31.12.2021	F		3	PPM	58	2	70	89	19	11	196	126	
09 *	03.01.2022	F		3	PPP	51	5	108	129	21	16	193	85	
10 *	05.01.2022	M		2	PP	49	4	93	281	188	7	191	98	
11 *	07.01.2022	F		3	PPP	51	3	66	74	8	10	189	123	
12	08.01.2022	M		2	JP	69	4	65	317	252	18	188	123	
13 *	11.01.2022	F		2	PP	54	7	62	264	202	13	185	123	
14 *	11.01.2022	F		2	MM	29	3	59	206	147	10	185	126	
15	19.01.2022	F		3	PPM	30	3	51	80	29	5	177	126	
16	21.01.2022	F		3	PPP	42	7	49	107	58	10	175	126	
17	07.02.2022	M		0	NA	47	9	35	NA	NA	13	158	123	
18 *	23.03.2022	F		3	MMP	50	7	112	211	99	16	114	2	
19 *	21.04.2022	F	B.1.1.529 Omicron BA.2	3	MMM	53	5	55	226	171	13	85	30	
20 *	02.05.2022	F		3	PPP	52	7	44	156	112	7	74	30	
			Delta = 6	0 = 2	P = 1, M = 1, J = 2, MMP = 2, PPP = 5, PPM = 2, PP = 2, JP = 1, MM = 1, MMM = 1, NA = 2	50	5	68	170	100	13	190	123	median
Frequency (n out of 20 Subjects)		M = 6	Omicron BA.1 = 12	1 = 2		18	1	35	74	4	5	74	2	min
		F = 14	Omicron BA.2 = 2	2 = 6		69	13	202	434	261	34	357	238	max
				3 = 10										

* HLA-A*02+ Subject; ^#^ P = Pfizer, M = Moderna, J = Johnson & Johnson; ^§^ PS = Positivity–Sampling; VS = Vaccine–Sampling; VP = Vaccine Positivity; PN = Positivity–Negativity; PCO = Positivity–Cut-off date; SCO = Sampling–Cut-off date; ΔT = Delta Timing (time lapse between the two events).

## Data Availability

The raw data supporting the conclusions of this article will be made available by the authors upon reasonable request, due to the complexity especially of flow cytometric data. However, most of the underlying experimental data are already resembled in the manuscript and its Supplementary Materials, where they are extensively displayed through representative plots, graphs, and detailed figure panels. In particular, the Supplementary Tables S1 and S2, and in Supplementary Figures S1, S3 and S5–S7 provide a comprehensive visualization of the datasets generated during the study.

## Supplementary Materials

The following supporting information can be downloaded at: https://www.mdpi.com/article/10.3390/ijms27146518/s1.

## Abbreviations

The following abbreviations are used in this manuscript:

%RD	% Residual DNA damage
%TI	% Tail Intensity/% DNA in Tail
AI	Artificial Intelligence
ASL RM1	Azienda Sanitaria Locale Roma 1
CDP	COVID-19 Data Portal
CEF	CMV-EBV-Influenza epitopes
CM	Central Memory
CMV	Cytomegalovirus
COI	Cut-Off Index
COVID-19	Coronavirus Disease 2019
DMSO	Dimethyl Sulfoxide
DN	Double-Negative
DNPH	2,4-Dinitrophenylhydrazine
DP	Double-Positive
EBV	Epstein–Barr Virus
EM	Effector Memory
FITC	Fluorescein Isothiocyanate
FLU	Influenza virus
Gr–Mo	Granulocyte–Monocyte doublets
HBV	Hepatitis B Virus
HCV	Hepatitis C Virus
HIV	Human Immunodeficiency Virus
HLA	Human Leukocyte Antigen
IAS	Image Analysis Software
IFN-γ	Interferon-gamma
ISS	Istituto Superiore di Sanità
MDA	Malondialdehyde
MFC	Multiparametric Flow Cytometry
MFI	Mean Fluorescence Intensity
NA	Not Applicable
NF-κB	Nuclear Factor kappa-light-chain-enhancer of activated B cells
NMR	Nuclear Magnetic Resonance
NP	Nucleocapsid Protein
PBMCs	Peripheral Blood Mononuclear Cells
PC/PCs	Principal Component(s)
PCA	Principal Component Analysis
PCO-ΔT	Calculated time intervals between Positivity and Cut-Off date
PD-L1	Programmed Death-Ligand 1
PN-ΔT	Calculated time intervals between Positivity and Negativity
PS-ΔT	Calculated time intervals between Positivity and Sampling
RBD	Receptor-Binding Domain
SARS-CoV-2	Severe Acute Respiratory Syndrome Coronavirus 2
SCO-ΔT	Calculated time intervals between Sampling and Cut-Off date
SEB	Staphylococcal Enterotoxin B
SFC	Spot-Forming Cell
SFCI	Spot-Forming Cell Index
SPSS	Statistical Package for the Social Sciences
TBA	Thiobarbituric Acid
TBARS	Thiobarbituric Acid Reactive Substances
TCA (1st use)	Tricarboxylic Acid (Krebs cycle context)
TCA (2nd use)	Trichloroacetic Acid (protein carboxylic acids protocol)
TD	Terminally Differentiated
VOC	Variant of Concern
VP-ΔT	Calculated time intervals between Vaccination and Positivity
VS-ΔT	Calculated time intervals between Vaccination and Sampling
