# Supplementary material for: Integrative Multidimensional Profiling of Individuals Recovered from Mild COVID-19 Reveals Immune–Metabolic–Oxidative Network Interactions
Source: Int J Mol Sci. 2026 Jul 22;27(14):6518. doi: 10.3390/ijms27146518 (PMC13409994; doi:10.3390/ijms27146518)
Supplement: Supplementary file 1 [file ijms-27-06518-s001.zip › ijms-4411902-supplementary.pdf]

Iole Macchia, Valentina La Sorsa, Francesca Marcon, Cristina Andreoli, Alessandro Giuliani, Donatella Pietraforte, Maria Cristina Quattrini, Egidio Iorio, Mattea Chirico, Maria Elena Pisanu, Enrica Montefiore, Francesca Luciani, Antonio Martina, Fabiola Mancini, Martina Borghi, Valentina Durastanti, Maria Concetta Altavista and Francesca Urbani.

Integrative multidimensional profiling of individuals recovered from mild COVID-19 reveals immune–metabolic–oxidative network interactions

Supplementary materials

Supplementary Table S1. Symptoms

Subj# ID	Malaise/fatigue	Fever	Headache	Cough	Throat pain	Rhinorrhea	Joint pain	Muscle pain	Ageusia	Anosmia	Confusional state	Shortness of breath	Thoracic pain	Rapid pulse	Diarrhea	Conjunctivitis	SpO2 < 96%	Vomiting nausea	Rash	Abdominal pain	Paresthesia	Dental mandibular pain
01	+	+	+	-	-	-	-	-	-	-	+	-	-	-	-	-	-	-	-	-	-	-
02	-	-	-	-	-	-	-	-	-	-	-	-	-	+	-	-	-	-	-	-	-	-
03	+	+	-	-	-	-	+	+	+	-	-	-	-	-	-	-	-	-	-	-	-	-
04	+	+	-	+	-	-	-	-	+	+	+	-	-	-	+	-	-	-	-	-	-	-
05	+	+	+	+	+	-	+	+	+	+	+	+	-	+	-	-	-	-	-	+	-	-
06	-	+	-	-	-	+	-	-	+	+	-	-	-	-	-	-	-	-	-	-	+	-
07	+	+	+	-	-	-	+	-	-	-	-	-	-	-	-	-	-	-	-	-	-	-
08	-	-	-	-	-	+	-	-	-	-	-	-	-	-	-	+	-	-	-	-	-	-
09	+	-	+	-	+	-	-	-	-	-	-	+	-	-	-	+	-	-	-	-	-	-
10	-	+	-	-	-	+	-	-	+	+	-	-	-	-	-	-	-	-	-	-	-	-
11	+	-	-	+	+	-	-	-	-	-	-	-	-	-	-	-	-	-	-	-	-	-
12	-	+	-	+	+	-	-	-	-	-	-	-	-	-	-	-	+	-	-	-	-	-
13	-	-	+	-	-	-	+	-	-	-	-	-	+	-	-	-	-	-	-	-	-	-
14	-	-	+	+	+	-	-	-	+	-	-	-	-	-	+	-	-	+	+	-	-	-
15	-	-	-	+	-	+	-	-	-	-	-	-	+	-	-	-	-	-	-	-	-	-
16	+	+	+	+	-	+	+	+	-	-	-	-	-	-	-	-	-	-	-	-	-	-
17	+	+	+	-	-	+	+	+	-	-	-	+	+	+	-	-	-	-	-	-	-	-
18	+	-	+	-	+	+	+	+	-	-	-	-	-	-	-	-	-	-	-	-	-	+
19	+	+	+	-	+	-	-	+	-	-	-	-	-	-	-	-	-	-	-	-	-	-
20	+	+	-	+	+	+	+	+	-	-	-	-	-	-	-	-	-	-	-	-	-	-
N =	12	12	10	8	8	8	8	7	6	4	3	3	3	3	2	2	1	1	1	1	1	1

No subjects showed any of the subsequent symptoms: Inability to walk, Wheezing, Bleeding, Lymphadenopathy, Seizures, Lower chest indrawing, Skin ulcer

Supplementary Table S2. Other clinical and lifestyle characteristics

Subj# ID	Concomitant therapy	Rx	Previous pathology	Flu vax	Paracetamol	Ibuprofen	Alcohol consumption	Allergy	Tobacco use	N_Acetylcysteine	Previous infectious disease	Intensive sport activity
1	+	+	+	-	+	-	-	+	-	-	+	+
2	+	-	+	-	-	-	-	-	-	-	+	-
3	-	-	-	-	-	-	-	-	+	-	-	-
4	+	+	-	-	-	-	-	-	-	+	-	-
5	+	+	-	-	-	+	-	-	-	-	-	-
6	-	-	-	-	-	-	+	-	-	-	-	-
7	-	+	-	+	-	-	-	-	-	-	-	-
8	+	-	-	+	+	-	-	-	-	-	-	-
9	+	+	-	-	-	+	-	-	-	-	-	-
10	+	+	-	+	-	-	-	-	+	-	-	-
11	+	+	+	+	+	-	+	-	-	-	-	-
12	-	-	-	+	-	-	+	-	+	-	-	-
13	+	-	-	-	+	+	+	-	-	-	-	+
14	-	-	-	-	-	-	-	-	-	-	-	+
15	+	+	+	+	-	+	-	-	-	-	-	-
16	+	+	+	+	+	-	-	+	-	-	-	-
17	+	-	+	-	-	+	-	+	+	+	-	-
18	+	+	+	+	-	-	-	-	-	+	-	-
19	-	+	-	-	-	-	-	-	-	-	-	-
20	+	-	+	-	-	-	-	+	-	-	+	-
N =	14	11	8	8	5	5	4	4	4	3	3	3

Supplementary Table S3. Flow Cytometry panel composition

	Staining panel	Antigen/Ligand	Fluorochrome	Clone	Species	Manufacturer	Nationality	Format
a)	Major CD3+ T cell and γδT cell memory/naive subsets	CD4	FITC	13B8,2	mouse	Beckman Coulter	California	Dried (Custom Tube)
		CCR7 (CD197)	PE	G043H7				
		CD8	Pe Cy5.5	B9.11				
		CD3	Pe Cy7	UCHT-1				
		CD45RA	APC	2H4				
		Vd2 TCR	APC-Fire 750	B6		Biolegend		Liquid
		CD45	APC-Cy7	2D1		Beckman Coulter		
b)	Granulocyte and monocyte subsets	CD294	FITC	BM16	mouse	Beckman Coulter	California	DuraClone IM Granulocytes
		CD16	ECD	3G8				
		CD33	PC5.5	D3HL 60.251				
		CD11B	PE Cy7	Bear1				
		CD274 (PD-L1)	APC	PDL1.3.1				
		Lineage (CD3 CD14 CD19 CD56)	APC A700	UCHT1, RMO52, J3-119,				
		CD62L	APC A750	DREG56				
		CD15	Pacific Blue	80H5				
		CD45	Krome Orange	J.33				

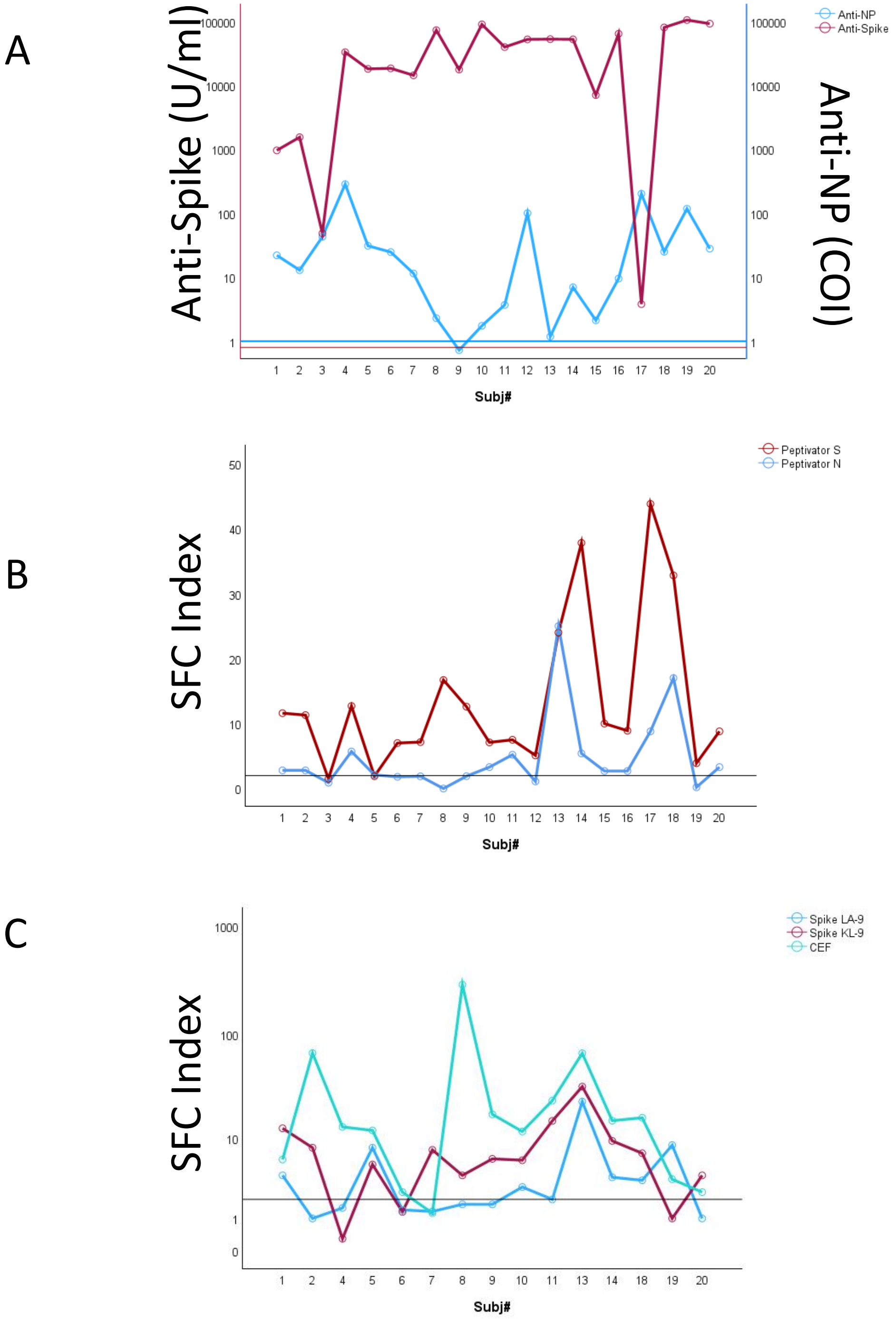

Supplementary Figure S1. Individual SARS-CoV-2–specific antibody titers and T cell responses. Line graphs depict individual values for each subject arranged chronologically by date of first positive SARS-CoV-2 test. (A) Anti-Spike (dark red, left y-axis, U/mL) and anti-NP (light blue, right y-axis, COI) antibody titers on a logarithmic scale Horizontal lines indicate the respective positivity thresholds (0.8 U/mL and 1 COI). (B) IFN-γ ELISpot responses to Peptivator S (light blue) and Peptivator N (dark red) peptide pools, expressed as Spot Forming Cell Index (SFCI), for all 20 subjects. (C) IFN-γ ELISpot responses to the HLA-A*02:01–restricted Spike peptides LA-9 (light blue) and KL-9 (dark red), and to the CEF positive control pool (cyan), expressed as SFCI, for 15 HLA-A*02:01–positive subjects. The positivity threshold for panels B and C is SFCI=2 (horizontal line).

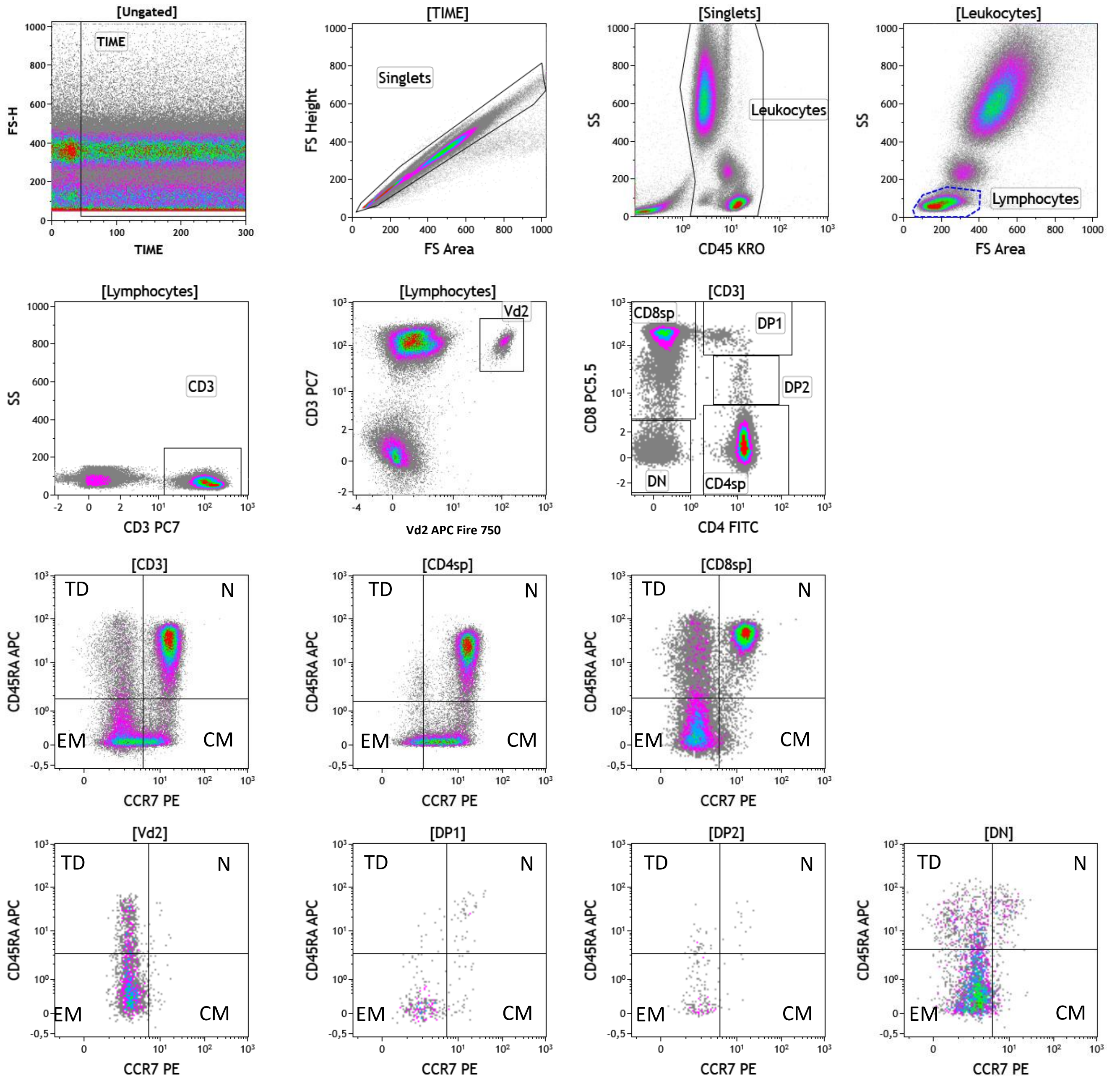

Supplementary Figure S2. Gating strategy for the naïve/memory T cell immunophenotyping panel. Representative dot plots illustrating the sequential gating strategy applied to identify CD3$^+$ T cell subsets and their maturation status in fresh whole blood. Whole blood samples were stained with a 7-color panel comprising anti-CD45, -CD45RA, -Vδ2, -CCR7, -CD8, -CD4, and -CD3 monoclonal antibodies. (A) Events with unstable acquisition kinetics, debris, and cell aggregates were excluded; sequentially, leukocytes were defined as CD45$^+$ cells and lymphocytes were identified within an SSC-A/FSC-A scatter plot. (B) Within the lymphocyte gate, CD3$^+$ and CD3$^+$TCRVδ2$^+$ γδ T cells were identified, while CD4sp, CD8sp, DP1 (CD8^{hi}CD4^{low}), DP2 (CD8^{low}CD4^{hi}), and DN (CD4$^-$CD8$^-$) subsets were in CD3$^+$ gate. (C) Within each subset, maturation status was defined based on CD45RA and CCR7 co-expression as naïve (N; CD45RA$^+$CCR7$^+$), central memory (CM; CD45RA$^-$CCR7$^+$), effector memory (EM; CD45RA$^-$CCR7$^-$), and terminally differentiated (TD; CD45RA$^+$CCR7$^-$).

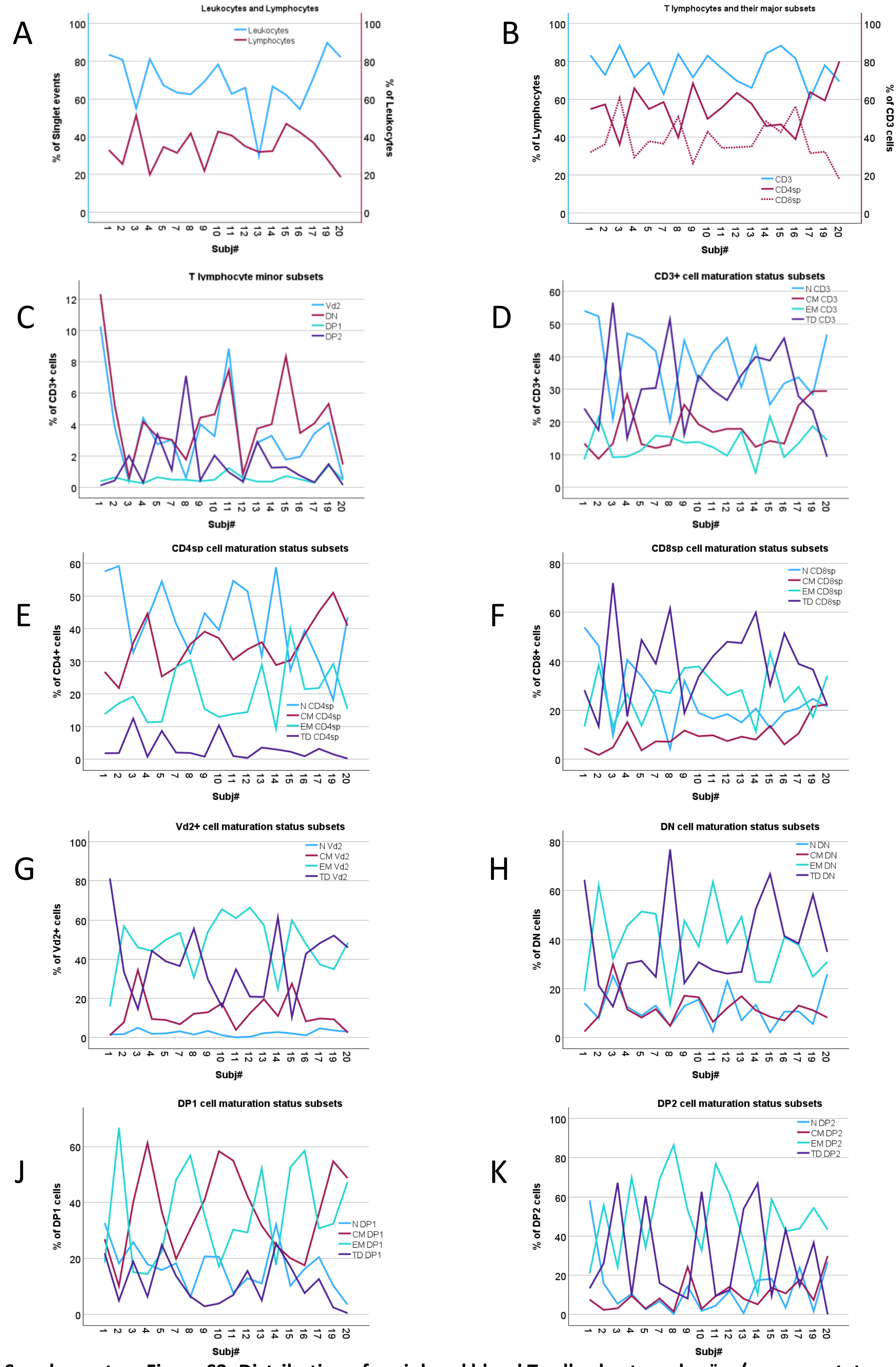

Supplementary Figure S3. Distribution of peripheral blood T cell subsets and naïve/memory status

Supplementary Figure S3. Distribution of peripheral blood T cell subsets and naïve/memory status
Line plots show the distribution of T cell subsets and their naïve/memory differentiation status across 18 subjects, with data expressed as percentages of the respective parent populations. Panel **A** shows leukocytes (blue line) as a percentage of singlet events and lymphocytes (red line) as a percentage of leukocytes. Panel **B** illustrates $CD3^+$ T cells in the lymphocyte gate and their subsets, including CD4 single-positive (CD4sp) and CD8 single-positive (CD8sp) cells, expressed as percentages of total $CD3^+$ T cells. Panel **C** reports minor $CD3^+$ T cell subsets ($V\delta 2^+$ γδ, DN, DP1, and DP2), expressed as percentages of total $CD3^+$ T cells. Panel **D** depicts the overall naïve (N), central memory (CM), effector memory (EM), and terminally differentiated (TD) distribution within total $CD3^+$ T cells, while Panels **E–K** show the naïve/memory composition within each T cell subset, with consistent color coding applied across all panels to facilitate comparison.

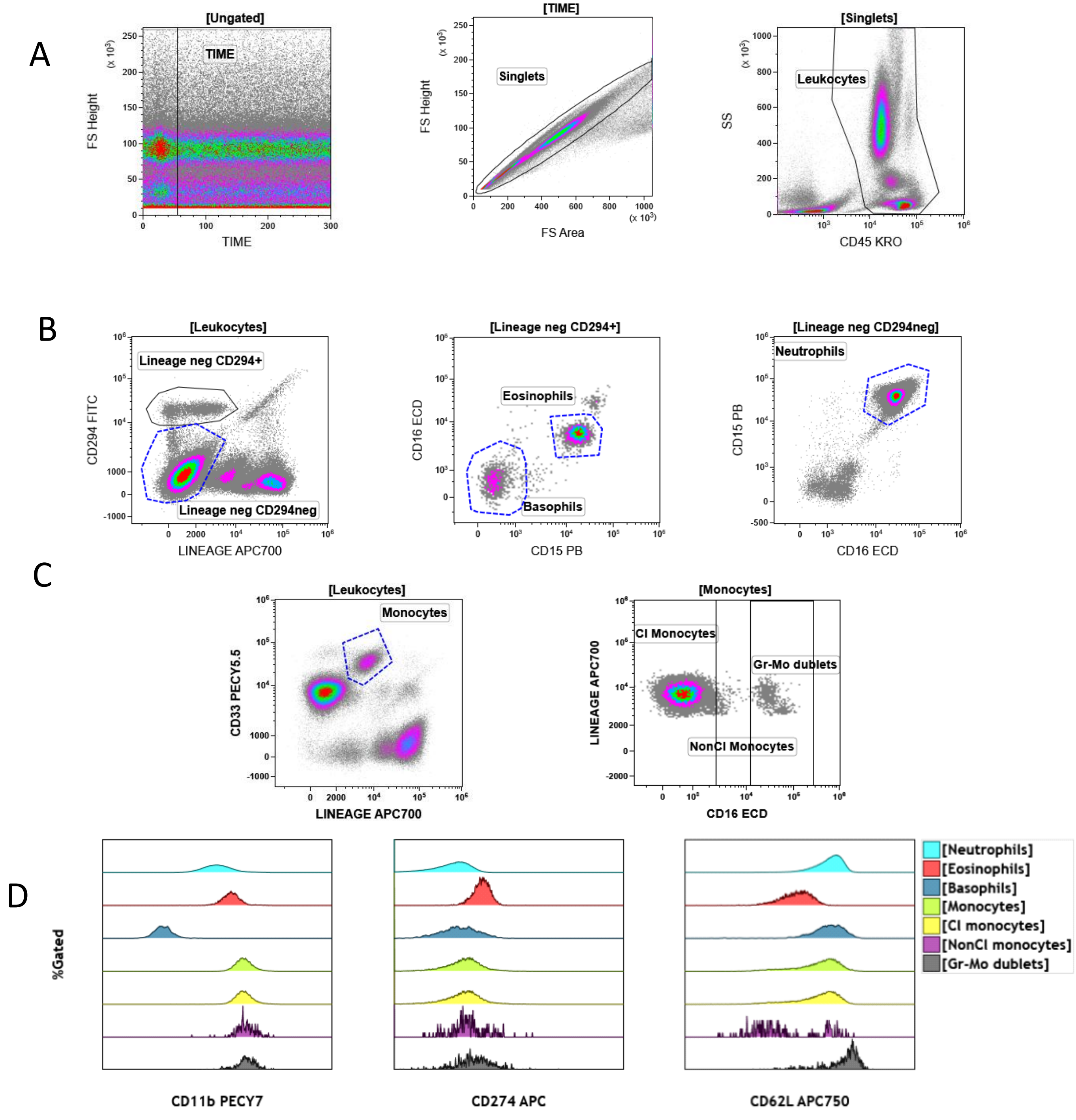

Supplementary Figure S4. Gating strategy for granulocyte and monocyte immunophenotyping. Representative dot plots illustrating the sequential gating strategy applied to identify granulocyte and monocyte subsets in fresh peripheral blood using a 10-color Duraclone panel. A) Events with unstable acquisition kinetics, debris, and cell aggregates were excluded sequentially; leukocytes were defined as $CD45^{+}$ cells. B) Within the leukocyte gate, neutrophils were identified as $CD15^{+}CD16^{+}$ lineage-negative ($CD3^{-}CD14^{-}CD56^{-}CD19^{-}$) $CD294^{-}$ cells. Within the lineage-negative $CD294^{+}$ gate, eosinophils ($CD15^{+}CD16^{int}$) and basophils ($CD15^{-}CD16^{-}$) were identified. C) Within the $CD33^{+}$ lineage-intermediate gate, monocytes were resolved into classical ($CD16^{-}$), non-classical ($CD16^{+}$), and Gr–Mo doublets ($CD16^{hi}$), the latter characterized by a granulocyte-like phenotype and reduced monocytic features. D) Surface expression of CD11b (activation marker), CD274/PD-L1 (immune checkpoint marker), and CD62L (adhesion molecule, downregulated upon cell activation) is reported as mean fluorescence intensity (MFI) for each subset.

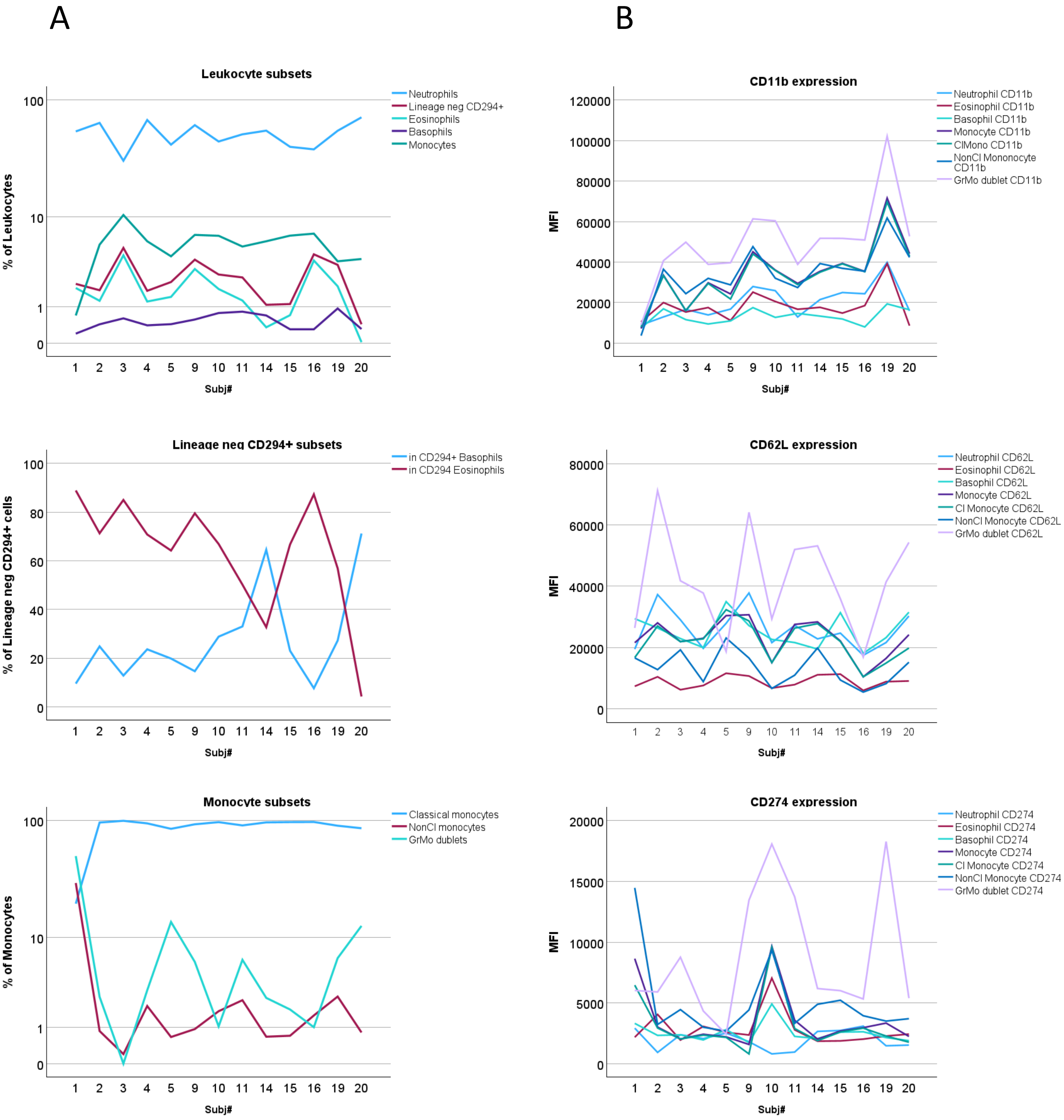

Supplementary Figure S5. Distribution of granulocyte and monocyte subsets and surface marker expression Line plots show the distribution of granulocyte and monocyte subsets and surface marker expression across 13 subjects, with data expressed as percentages of the respective parent populations or as mean fluorescence intensity (MFI), as appropriate. Panel **A** illustrates the frequency of granulocyte and monocyte subsets, including: neutrophils, eosinophils, basophils; CD294+ eosinophils and CD294+ basophils; classical monocytes, non-classical monocytes, and Gr–Mo doublets, all expressed as percentages of the relevant parent gate. Panel **B** shows surface expression of CD11b, CD274/PD-L1, and CD62L, reported as MFI for each subset, with consistent color coding applied across panels to facilitate comparison.

A

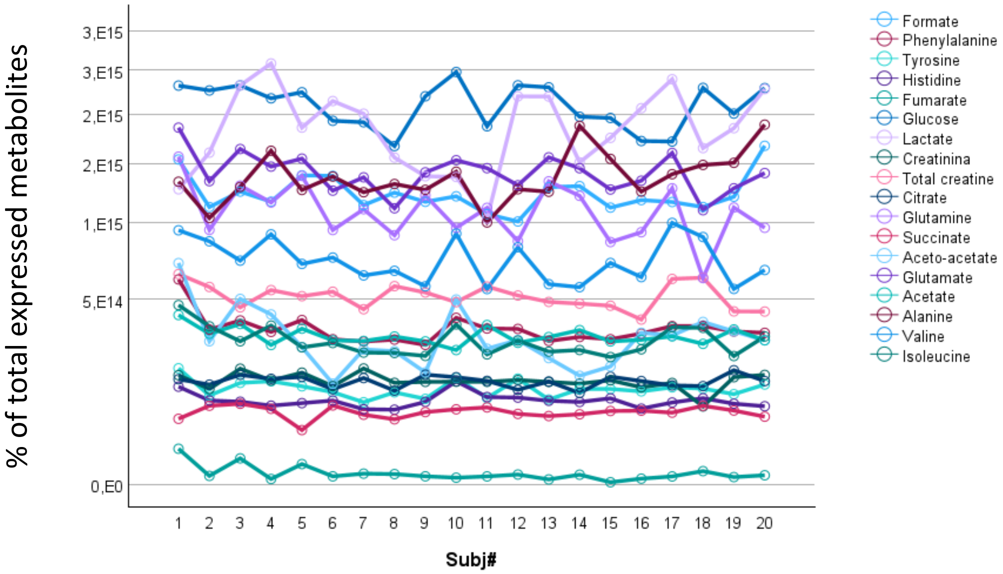

B

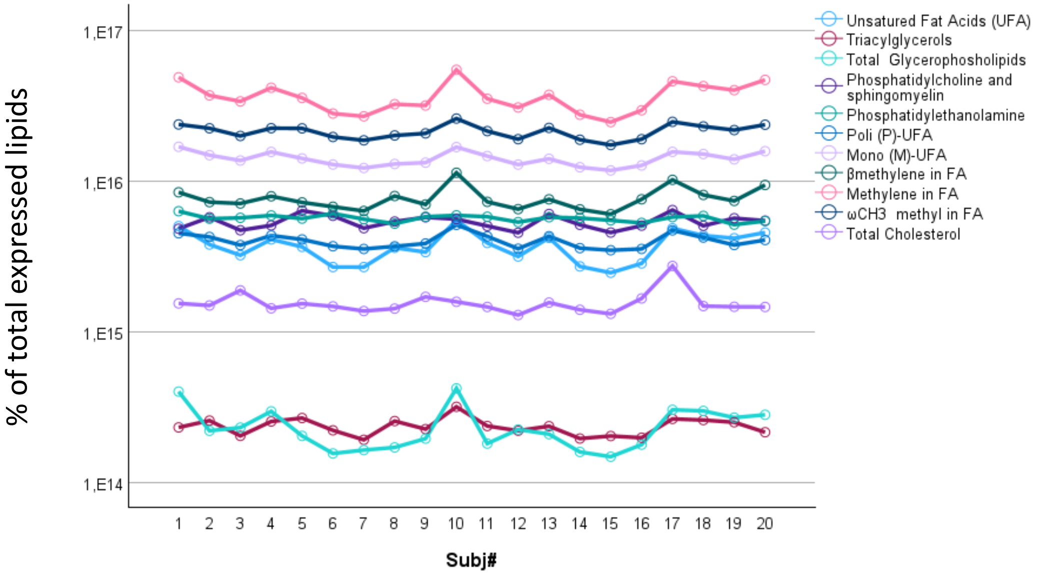

Supplementary Figure S6. Individual plasma metabolomic and lipidomic profiles. Line graphs display normalized and scaled values for each detected analyte across all subjects, arranged chronologically by date of first positive SARS-CoV-2 test. (A) Individual plasma metabolites quantified by NMR spectroscopy. (B) Individual plasma lipid species quantified by NMR spectroscopy. The visual complexity of both panels reflects the high dimensionality of the datasets, which motivated dimensionality reduction by PCA (see Figure 3).

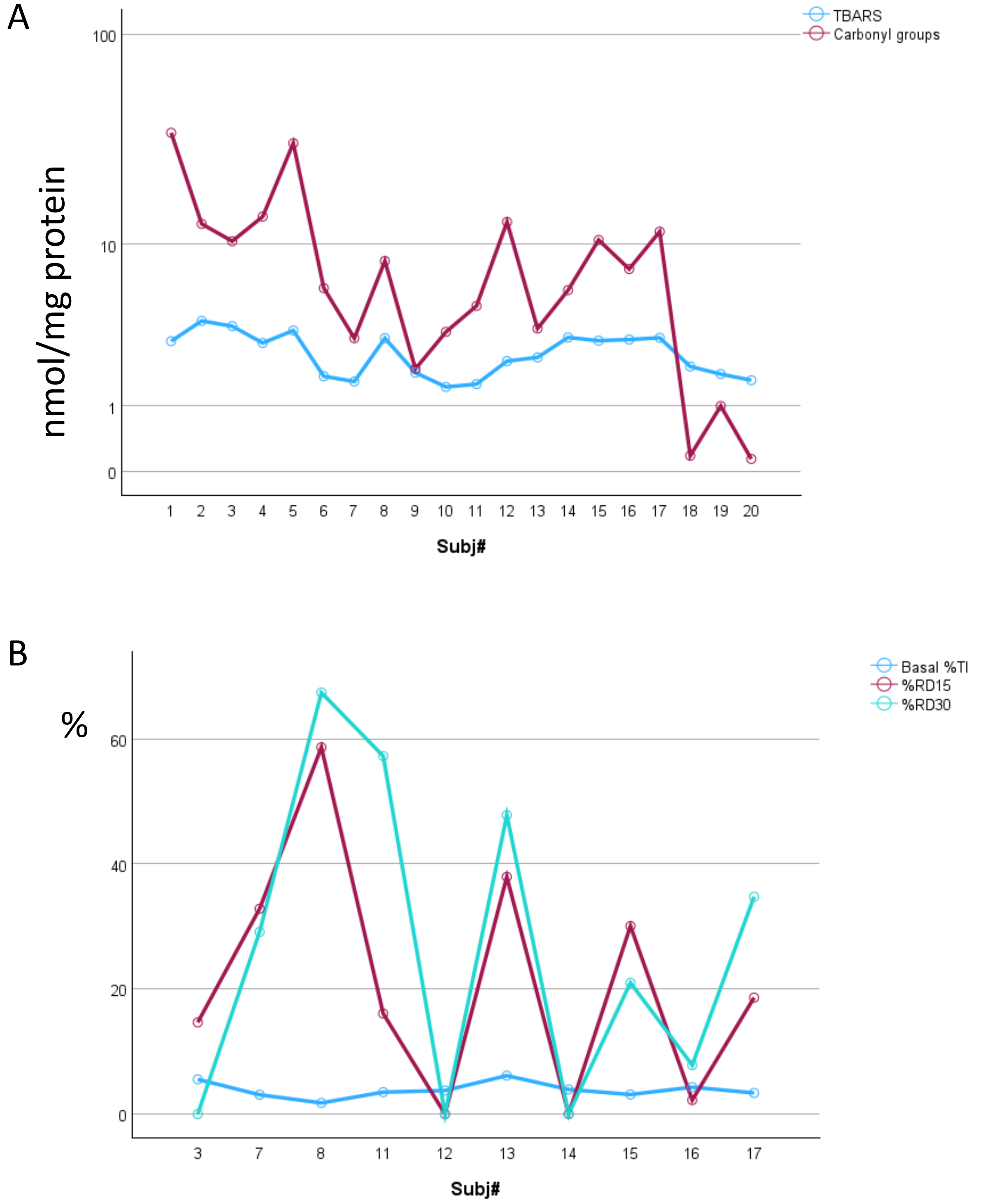

Supplementary Figure S7. Individual plasma oxidative stress levels and DNA damage parameters. Line graphs show individual values arranged chronologically by date of first positive SARS-CoV-2 test. (A) Plasma TBARS (reflecting lipid peroxidation; dark red) and protein carbonyl groups (reflecting protein oxidation; light blue) across all 20 enrolled subjects. (B) DNA damage and repair parameters — %RD15, %RD30 (residual DNA damage at 15 and 30 minutes post-irradiation, reflecting DNA repair capacity), and %TI (tail intensity at baseline, reflecting basal DNA strand break levels) across a subgroup of 10 subjects analyzed.

Supplementary Table S4. Sensitivity analysis of the correlation network: cluster-stratified FDR correction

Variable 1	Variable 2	Family (cluster1 / cluster2)	Family m	Rho	N	Raw p-value	m based FDR-BH adjusted p-value (q)
VP-ΔT	VS-ΔT	Time lapse / Time lapse	15	0.874	18	0.00000	0.00003
Carbonyl groups	SCO-ΔT	Time lapse / Oxidative stress	12	0.828	20	0.00001	0.00008
Metabolite PC1	Vaccine dose number	Vaccine/VOC / Metabolite PC	12	-0.785	20	0.00004	0.00050
PCO-ΔT	Delta Omicron	Vaccine/VOC / Time lapse	18	-0.795	20	0.00003	0.00051
TBARS	SCO-ΔT	Time lapse / Oxidative stress	12	0.724	20	0.00031	0.00187
Sex	Tobacco use	Demographic / Clinics / Life style	24	-0.764	20	0.00009	0.00213
CEF	Fever	Cellular response / Symptoms	75	-0.867	15	0.00003	0.00217
Carbonyl groups	TBARS	Oxidative stress / Oxidative stress	1	0.642	20	0.00227	0.00227
PCO-ΔT	PS-ΔT	Time lapse / Time lapse	15	0.693	20	0.00071	0.00533
Metabolite PC1	Lipid PC1	Lipid PC / Metabolite PC	8	0.693	20	0.00070	0.00561
Carbonyl groups	Vaccine dose number	Vaccine/VOC / Oxidative stress	6	-0.678	20	0.00102	0.00615
Anosmia	Ageusia	Symptoms / Symptoms	105	0.764	20	0.00009	0.00934
Carbonyl groups	Delta Omicron	Vaccine/VOC / Oxidative stress	6	-0.624	20	0.00325	0.00975
Granulocyte PC2	PCO-ΔT	Time lapse / Granulocyte PC	12	0.808	13	0.00084	0.01007
TBARS	Vaccine dose number	Vaccine/VOC / Oxidative stress	6	-0.6	20	0.00517	0.01034
SCO-ΔT	PCO-ΔT	Time lapse / Time lapse	15	0.634	20	0.00266	0.01331
%RD30	%RD15	DNA damage / DNA damage	3	0.806	10	0.00485	0.01455
Granulocyte PC2	Delta Omicron	Vaccine/VOC / Granulocyte PC	6	-0.761	13	0.00254	0.01522
Granulocyte PC2	Anti-Spike	Granulocyte PC / Antibody response	4	-0.736	13	0.00411	0.01643
Carbonyl groups	Anti-Spike	Antibody response / Oxidative stress	4	-0.609	20	0.00437	0.01748
Granulocyte PC2	Vaccine dose number	Vaccine/VOC / Granulocyte PC	6	-0.714	13	0.00609	0.01828
Metabolite PC3	%RD30	DNA damage / Metabolite PC	12	-0.853	10	0.00171	0.02054
Carbonyl groups	PCO-ΔT	Time lapse / Oxidative stress	12	0.599	20	0.00528	0.02114
Age	Throath pain	Demographic / Symptoms	30	0.692	20	0.00073	0.02193
Peptivator N	Peptivator S	Cellular response / Cellular response	10	0.644	20	0.00220	0.02201
Delta Omicron	Vaccine dose number	Vaccine/VOC / Vaccine/VOC	3	0.571	20	0.00851	0.02554
Joint pain	Muscle pain	Symptoms / Symptoms	105	0.685	20	0.00087	0.03845
Total symptoms	Muscle pain	Symptoms / Symptoms	105	0.675	20	0.00110	0.03845
Ibuprofen	Shortness of breath	Symptoms / Clinics / Life style	180	0.728	20	0.00028	0.04986
Granulocyte PC2	Carbonyl groups	Granulocyte PC / Oxidative stress	4	0.665	13	0.01317	0.05266
SCO-ΔT	Vaccine dose number	Vaccine/VOC / Time lapse	18	-0.58	20	0.00735	0.06380
SCO-ΔT	Delta Omicron	Vaccine/VOC / Time lapse	18	-0.558	20	0.01063	0.06380
Anti-Spike	Vaccine dose number	Vaccine/VOC / Antibody response	6	0.542	20	0.01366	0.06401
Metabolite PC3	VP-ΔT	Time lapse / Metabolite PC	24	0.662	18	0.00279	0.06700
Anti-NP	Fever	Antibody response / Symptoms	30	0.637	20	0.00251	0.07541
Anti-Spike	PCO-ΔT	Time lapse / Antibody response	12	-0.581	20	0.00717	0.08604
Confusional state	Delta Omicron	Vaccine/VOC / Symptoms	45	-0.642	20	0.00229	0.10304
Carbonyl groups	Confusional state	Symptoms / Oxidative stress	30	0.619	20	0.00360	0.10792
Lipid PC2	Shortness of breath	Lipid PC / Symptoms	30	0.619	20	0.00360	0.10792
Rapid pulse	Shortness of breath	Symptoms / Symptoms	105	0.608	20	0.00447	0.11012
Muscle pain	Malaise Fatigue	Symptoms / Symptoms	105	0.599	20	0.00524	0.11012
Lipid PC2	Ibuprofen	Lipid PC / Clinics / Life style	24	0.551	20	0.01186	0.14674
Lipid PC1	Concomitant therapy	Lipid PC / Clinics / Life style	24	0.549	20	0.01223	0.14674
VS-ΔT	Sex	Demographic / Time lapse	12	-0.567	18	0.01415	0.16976
T cells PC4	Ageusia	T cell PC / Symptoms	60	-0.634	18	0.00473	0.18782
T cells PC4	Anosmia	T cell PC / Symptoms	60	-0.618	18	0.00626	0.18782
TBARS	Rapid pulse	Symptoms / Oxidative stress	30	0.546	20	0.01268	0.19021
T cells PC2	Spike LA9	T cell PC / Cellular response	20	0.686	13	0.00964	0.19272
Total symptoms	Malaise Fatigue	Symptoms / Symptoms	105	0.549	20	0.01218	0.21311
Anti-NP	Muscle pain	Antibody response / Symptoms	30	0.536	20	0.01478	0.22175
Rapid pulse	Vaccine dose number	Vaccine/VOC / Symptoms	45	-0.55	20	0.01202	0.26647
Previous pathology	Allergy	Clinics / Life style / Clinics / Life style	66	0.612	20	0.00410	0.27081
Spike LA9	Headache	Cellular response / Symptoms	75	0.635	15	0.01095	0.30590
Peptivator S	Fever	Cellular response / Symptoms	75	-0.549	20	0.01224	0.30590
Peptivator N	N-Acetylcysteine	Cellular response / Clinics / Life style	60	0.547	20	0.01256	0.31747
Spike KL9	Intensive sport activity	Cellular response / Clinics / Life style	60	0.618	15	0.01412	0.31747
SCO-ΔT	Confusional state	Time lapse / Symptoms	90	0.568	20	0.00905	0.31933
PS-ΔT	Cough	Time lapse / Symptoms	90	-0.566	20	0.00923	0.31933
VP-ΔT	Ageusia	Time lapse / Symptoms	90	0.586	18	0.01064	0.31933

q<=0.015 (orange, n=17/59); q<=0.050 (orange+yellow, n=29/59); q<=0.10 (orange+yellow+green, n=36/59).

Supplementary Table S5

T cell PCA - SCORE COEFFICIENTS

	PC1	PC2	PC3	PC4
Leukocytes	2.18	-1.08	1.45	-0.37
Lymphocytes	0.39	1.07	-0.31	0.63
CD3	2.58	1.02	0.51	0.35
CM CD3	-0.44	-0.62	-0.03	-0.24
N CD3	0.62	-0.90	0.63	-1.19
EM CD3	-0.69	-0.28	-0.55	0.33
TD CD3	0.19	1.73	-0.26	1.02
CD4sp	1.57	-1.31	-0.09	-0.94
N CD4sp	0.81	-0.22	0.72	-1.28
CM CD4sp	0.46	-0.20	-0.07	0.11
EM CD4sp	-0.35	0.08	-0.79	1.36
TD CD4sp	-1.24	0.26	-0.07	-0.27
CD8sp	0.59	1.51	-0.08	0.58
N CD8sp	-0.13	-1.00	1.07	-1.04
CM CD8sp	-0.88	-0.51	-0.11	0.18
EM CD8sp	0.04	-0.75	-1.23	0.19
TD CD8sp	0.67	2.19	0.06	0.60
Vδ2 γδ CD3	-1.23	-0.27	0.11	-0.06
N Vδ2 γδ	-1.28	-0.13	-0.04	0.01
CM Vδ2 γδ	-0.77	0.74	-0.68	0.09
EM Vδ2 γδ	1.16	0.04	-2.26	-1.10
TD Vδ2 γδ	0.58	-0.73	2.77	0.92
DP1	-1.37	-0.16	-0.10	0.03
N DP1	-0.53	0.30	0.88	-0.54
CM DP1	0.53	-0.43	-0.05	-1.34
EM DP1	0.52	-0.39	-1.69	1.82
TD DP1	-0.83	0.46	0.65	-0.01
DP2	-1.33	-0.03	-0.13	0.15
N DP2	-0.77	-1.05	1.60	0.24
CM DP2	-0.86	-0.88	-0.22	-0.03
EM DP2	1.20	-1.51	-2.10	1.48
TD DP2	0.11	3.37	0.52	-1.76
DN	-1.18	-0.25	0.10	0.13
N DN	-0.77	-0.07	0.16	-0.69
CM DN	-0.79	0.35	-0.42	-0.57
EM DN	0.68	-0.35	-1.41	-1.86
TD DN	0.56	0.00	1.46	3.05

Supplementary Table S6

Granulocyte PCA - SCORE COEFFICIENTS

var	PC1	PC2
neutrophils	-0.87	-0.29
neutrophil CD11b	0.50	-0.79
neutrophil CD274	-0.73	-0.11
neutrophil CD62L	0.96	1.33
lineage neg CD294+	-0.88	-0.29
in CD294+ eosinophils	-0.87	-0.29
in CD294+ basophils	-0.88	-0.29
eosinophils	-0.88	-0.29
eosinophil CD11b	0.35	-0.29
eosinophil CD274	-0.68	-0.21
eosinophil CD62L	-0.25	0.24
basophils	-0.88	-0.29
basophil CD11b	0.02	0.10
basophil CD274	-0.69	-0.11
basophil CD62L	0.94	1.91
monocytes	-0.88	-0.29
monocyte CD11b	1.43	-1.56
monocyte CD274	-0.61	0.24
monocyte CD62L	0.78	1.78
Classical monocytes	-0.87	-0.29
Cl monocyte CD11b	1.39	-1.50
C lmonocyte CD274	-0.64	0.07
Cl monocyte CD62L	0.71	1.40
NCl monocytes	-0.88	-0.29
NonCl monocyte CD11b	1.45	-1.45
NonCl monocyte CD274	-0.50	0.72
NonCl monocyte CD62L	0.09	1.41
GrMo doublets	-0.88	-0.29
GrMo doublet CD11b	2.49	-2.05
GrMo doublet CD274	-0.28	-0.24
GrMo doublet CD62L	2.02	2.03
